# Identifying functional regulatory mutation blocks by integrating genome sequencing and transcriptome data

**DOI:** 10.1016/j.isci.2023.107266

**Published:** 2023-07-03

**Authors:** Mingyi Yang, Omer Ali, Magnar Bjørås, Junbai Wang

**Affiliations:** 1 Department of Microbiology, Oslo University Hospital and University of Oslo, Oslo, Norway; 2 Department of Pathology, Oslo University Hospital - Norwegian Radium Hospital, Oslo, Norway; 3 Department of Medical Biochemistry, Oslo University Hospital and University of Oslo, Oslo, Norway; 4 Department of Clinical Molecular Biology (EpiGen), Akershus University Hospital and University of Oslo, Lørenskog, Norway; 5 Department of Clinical and Molecular Medicine, Norwegian University of Science and Technology, Trondheim, Norway; 6 Faculty of Medicine, University of Oslo, Oslo, Norway

**Keywords:** Biological sciences, Bioinformatics, Biocomputational method, Omics, Biological sciences tools

## Abstract

Millions of single nucleotide variants (SNVs) exist in the human genome; however, it remains challenging to identify functional SNVs associated with diseases. We propose a non-encoding SNVs analysis tool bpb3, BayesPI-BAR version 3, aiming to identify the functional mutation blocks (FMBs) by integrating genome sequencing and transcriptome data. The identified FMBs display high frequency SNVs, significant changes in transcription factors (TFs) binding affinity and are nearby the regulatory regions of differentially expressed genes. A two-level Bayesian approach with a biophysical model for protein-DNA interactions is implemented, to compute TF-DNA binding affinity changes based on clustered position weight matrices (PWMs) from over 1700 TF-motifs. The epigenetic data, such as the DNA methylome can also be integrated to scan FMBs. By testing the datasets from follicular lymphoma and melanoma, bpb3 automatically and robustly identifies FMBs, demonstrating that bpb3 can provide insight into patho-mechanisms, and therapeutic targets from transcriptomic and genomic data.

## Highlights

• Integration of genome sequencing and transcriptome data for regulatory SNP analysis
• A Bayesian approach with a biophysical model to estimate TF-DNA binding affinity
• Use of DNA methylome data for identification of functional regulatory mutations
• The tool robustly identifies functional non-coding mutation regions in diseases

## Graphical abstract

**Figure:**
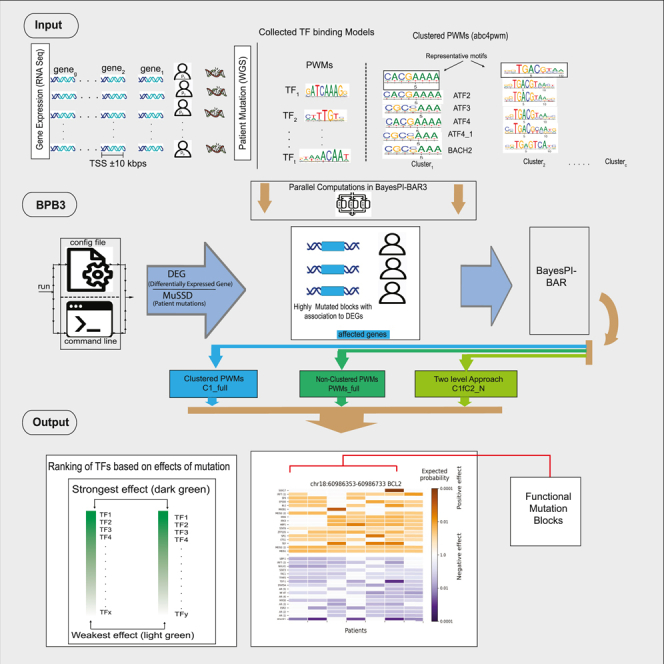

## Introduction

Genomic mutations and genetic variations often lead to dysregulation of gene expression and contribute to many diseases such as cancer, diabetes, and asthma.^1^^,^^2^ Mutations are changes in the DNA sequence that occur as a result of errors during DNA replication, exposure to mutagens (e.g., radiation, chemicals), or other genetic or environmental factors. Mutations can involve changes in one or more nucleotides (e.g., a substitution, insertion, or deletion). Genetic variation, on the other hand, refers to the natural differences that exist between individuals in a population due to variations in their DNA sequence. Genetic variation arises from a combination of different sources, including mutations, genetic recombination, and migration of individuals between populations. Genetic variation can be quantified at different levels, such as nucleotide, gene, or chromosome level. Single nucleotide polymorphisms (SNPs) are a type of genetic variation in nucleotide level, which occurs when a single nucleotide at particular location in the DNA sequence is different between individuals or populations. In this paper, we focus on the impact of substitution mutations or SNPs on transcription regulations. Since some SNPs are caused by substitution mutations, we hereafter call both mutations.

As high throughput sequencing techniques are becoming cheaper, more sequences data are being produced from various patient groups, which are available for disease diagnosis and understanding the molecular mechanisms. These large amounts of sequencing data need to be analyzed and explored. Especially, identifying the functional mutation regions from millions of genetic variants in the individual patient genome is an emerging task for understanding patho-mechanisms leading to disease, clustering patients, providing potential diagnostic biomarkers and screening of therapy targets.^3^^,^^4^^,^^5^ Gene expression is regulated at several levels and by numerous proteins, including DNA-binding transcription factors (TFs) facilitating activation or repression of transcription. Thousands of TFs have been identified in organisms from bacteria to human. TFs usually contains a conserved DNA binding domain (DBD), which recognizes conserved short DNA sequences (motif) or transcription factor binding sites (TFBSs) in the genome. Many studies show multiple genetic disorders caused by the disruption of TFBSs.^2^^,^^3^^,^^4^^,^^5^^,^^6^^,^^7^^,^^8^ These disruptions cause miss-recognition of the binding site of a transcription factor (TF). Consequently, the TF may attach to a different target sequence, or a new binding site is created by the mutation. Usually, the variations of gene expression profiles can indicate the effects of regulatory sequence variations. Several types of cancer (such as lung and breast cancer) are usually classified and predicted using these gene expression profiles.^9^^,^^10^

Genetic variants, such as somatic single nucleotide variants (SNVs) including SNPs, structural variants, insertions, and deletions, can affect the binding affinity of DNA with TFs, and are considered as a significant mechanism in the development of cancer or other diseases.^11^ Therefore, a computational model was proposed assuming that the alteration of protein-DNA interaction is the primary cause leading to dysregulation of gene expression by non-coding variants.^12^^,^^13^ This model uses position weight matrices (PWMs) to quantify TF-DNA binding affinity and estimate the effect of a given SNV. Recently collaborative work from The Cancer Genome Atlas (TCGA) and the International Cancer Genome Consortium (ICGC) revealed millions of SNVs in different types of cancers.^14^^,^^15^ Often, studies^16^^,^^17^ focus on selected few SNVs, and use a collection of TF-DNA binding sites to model against those SNVs. For every SNV, a list of possibly affected TF binding sites are predicted and sorted by impact or certainty. These SNVs in the non-coding regions of the genome not only affect binding affinity for TFs, but also for RNA-binding proteins (RBPs), and micro RNAs (miRNAs),^18^ which in turn affects multiple biological processes such as transcription, DNA methylation, mRNA translation, and chromatin structure.

Recently, whole genome sequencing technologies have enabled new discoveries in the medical field, including genome-wide profiling of mutations by scanning SNVs in both coding and non-coding regions. Several attempts are made to identify mutations affecting gene regulation, such as the mutations in cis regulatory elements (CREs), in a large number of cancer or disease datasets.^19^^,^^20^ The mutations and SNVs in CREs, including promoters, enhancers, and silencers in no-coding regions, play crucial roles in many types of disease and lead to dysregulation of gene expression.^4^^,^^5^^,^^21^ To search and annotate the functional mutations, several tools have been developed. For example, the FunSeq2 is an online tool and applied to annotate disease-causing SNVs or SNPs in a sequence variant file (in bed format) or variant calling file (in VCF format).^22^^,^^23^ The other similar tools include ANNOVAR,^24^ snpEff,^25^ Variant Effect Predictor (VEP)^26^ and VarScan.^27^ So far, several SNVs analysis tools have been developed using machine learning (ML) methods, which train models with large source datasets and predicts the impact of SNVs on disease in various tissue.^28^ Some of them use supervised methods by labeling sample phenotype in the training set, such as combined annotation-dependent depletion (CADD),^29^ deep neural network (DNN),^30^ linear INSIGHT (LINSIGHT)^31^ and non-coding essential regulation (ncER).^32^ Others use un-supervised methods without sample labeling, e.g., context-dependent tolerance score (CDTS)^33^ and Eigen.^34^ There are various statistical models implemented in these tools, including logistic regression (CADD), DNN, gradient-boosted decision trees (ncER), spectral meta-learner (Eigen), the combination of generalized linear regression model from functional genome data and a probabilistic model of molecular evolution (LINSIGHT). Usually, these approaches integrate many genomic features into a single score (e.g., combined score in CADD, Eigen score in Eigen) for each SNV. The score correlated with allelic diversity, function annotation, disease severity, pathogenic and regulatory effects from experiments measurement or/and protein evolution. These single-functional-score based methods are powerful for incorporating diverse information of an SNV when looking for key SNVs in disease.

However, one challenge in the aforementioned tools is that the information of gene expression and epigenetic profiles from patients are not considered, which makes them less useful when correlating SNVs with patient-specific omics data. Currently, only few studies integrated patient-specific whole genome sequencing data with gene expression data,^4^^,^^35^ more tools are needed to accomplish such kind of integrated data analysis. The second challenge is to efficiently identify hotspots of SNVs in genome-wide that would form functional clustering (e.g., functional mutation block—FMB) or region. Though few studies have addressed this issue: for example, an exact binomial test was used to search for enriched mutations by comparing mutations in an interesting region (e.g., a 5 kb window sliding at CRE site) with a background mutation rate based on Poisson model.^6^ Our understanding of functional regulatory mutations in cancer or disease development and progression is still limited, due to a lack of effective tools to study them.^21^ To address the aforementioned challenges, we present a package “bpb3”, BayesPI-BAR version 3, which is applied to identify the functional mutation blocks in no-coding SNVs regions by integrating genome sequencing and transcriptome data and/or user-defined regions generated from other omics datasets. New features of bpb3 include the incorporation of transcriptional data and/or information from other functional genomic data, and the application of clustered PWMs to speed up the calculation for TF binding affinity changes. It not only can be used to identify functional regulatory mutation-gene associations in cancers or disease, but also can be applied to search for therapeutic target. A detailed documentation, a series of demos and a user guide tutorial of bpb3 package are available at online (https://bpb3.github.io/bpb3/).

## Results

### Overview of the proposed bpb3 tool

**Figure 1:**
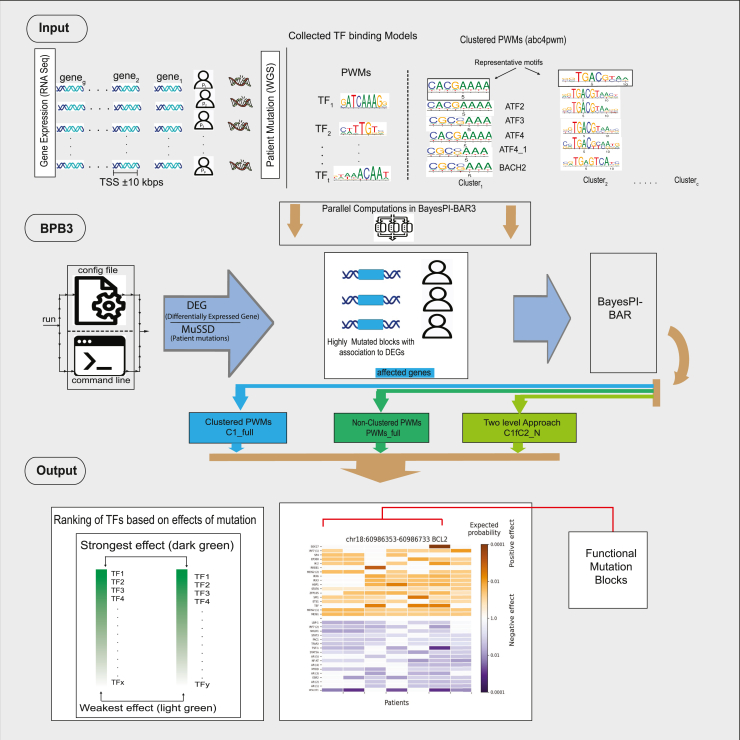
Overview of the proposed bpb3 package This figure illustrates the workflow of the BayesPI-Bar3 (bpb3) package. It predicts putative functional mutation blocks (FMBs) in cancer/disease based on both gene expression profiles and genome wide sequencing (e.g., SNPs) information from patients. Bpb3 predicts FMBs with high frequency in patients (e.g., MuSSD) and significantly affect TF binding affinity changes (e.g., BayesPI-BAR), in either predefined regions (e.g., ±10kbps to TSS of differential expression gene—DEG) or genome wide, by using a set of Position Weight Matrices (PWMs) of TFs. All functions of bpb3 can be run as command line application manually or automatically with a predefined configured file by modifying the corresponding parameters for different tasks. There are three types of methods in bpb3 to predict significant TF-DNA binding affinity changes due to patient-specific mutations: 1) Clustered PWMs—C1_full; 2) All PWMs—PWMs_full; 3) Two level method by selecting PWMs from the top N ranked clusters from C1_full—C1fC2_N. Bpb3 supports parallel computation. It outputs a list of top-ranked TFs with significant TF binding affinity changes caused by FMBs and illustrates them in a color code heatmap.

A Bayesian method similar to BayesPI-BAR2^36^ is implemented in bpb3 for computing the protein-DNA interaction with binding affinity ranking. We provide over 1700 built-in human PWMs to quantify the TF-DNA binding affinity and estimate the impact on a given sequence variant region. Furthermore, bpb3 provides a substantial update and functional expansion of the tool (Figure 1). It is a user-friendly python3 package with a clustered PWMs module for speeding up omics data integration. For a simple functional mutation identification, it requires to input both the alternative DNA sequence (contain SNPs) and the corresponding reference sequence. For an integrated data analyses of mutations, both the genome sequences (e.g., alternative and reference sequences) and the gene expression profiles (e.g., count matrix file from RNA-seq) of patients and controls are needed. Alternatively, bpb3 can integrate data with pre-defined regions such as differential methylation regions (DMR) predicted from DNA methylome data. In output, each statistically significant functional mutation block (FMB) can be visualized with a color coded heatmap, where detailed information are provided (e.g., chromosome position, mutation distribution in patients and the top-ranked TFs with significant TF-DNA binding affinity changes). Finally, a precompiled configuration file, with easily editable paths and parameters, is provided for user to run bpb3 automatically. Modules for pre-processing of data downloaded from ICGC and cleaning up temporary files are also part of the bpb3 package.

### Validation of prediction accuracy based on 67 known regulatory SNPs

Here, ∼67 SNPs^1^^,^^37^^,^^38^ that are known to affect TF binding in the regulatory regions were used to evaluate the bpb3 package. These SNPs were used successfully in previous publications^36^^,^^39^ as a golden standard to evaluate the accuracy of *in silico* prediction of TF binding affinity changes due to DNA sequence variation. All calculations were done using 10 parallel processes on the same high-performance computer. The clustering of 1772 human TFs was obtained by applying the abc4pwm package^40^ within each pre-classified DBD family. To predict TF binding affinity changes, three types of calculations were used: (1) calculation based on all 1772 human non-classified TFs (named as PWMs_full method); (2) calculation using 1772 clustered human TFs based on pre-classified DBD family including ∼736 pseudo-human (or representative) TFs (Clustered PWMs level 1, shortly named as C1_full method); (3) calculation using selected PWMs from the top-ranked clusters or pseudo-TFs from C1_full (Clustered PWMs level 2, shortly named as C2 method or C1fC2, the two-level approach). The prediction accuracy of each method is computed by counting the number of TF targets, recovered in the top N-ranked TFs or pseudo-TFs (shortly named as C2_N or C1fC2_N in test, e.g., C2_20 recovered in the top 20 TFs), belonging to the verified TF targets of SNPs. The results suggest that the prediction accuracy of exported top 15 to 25 TFs in C2 is about ∼80% based on the top 20 to 25 pseudo-human TFs from clustered PWMs (C1_full). This result is close to or better than the prediction accuracy achieved using all 1772 TFs (PWMs_full); refer to Table 1 for the validation of the results. Obviously, the computational times needed for C1_full (∼12 min) and C2 (e.g., ∼9 min C2_20) are much less than that for the full calculation (PWMs_full; ∼30 min). Please refer to Table 1 and Figure S1. Thus, the C1_full method not only gives good prediction accuracy, but also speeds up the whole calculation.

**Table 1:** Prediction accuracy of TF binding affinity changes for 67 known regulatory SNPs based on three types of calculations in bpb3 package

Method	Elapsed time (minutes)	Top10	Top15	Top20	Top25	Top30	Top40
PWMs_full	31	73%	76%	79%	85%	**88%**	**88%**
C1_full	12	77.6%	83.6%	88%	**89.6%**	**89.6%**	**89.6%**
C2_10	5	74.6%	**77.6%**	**77.6%**	**77.6%**	**77.6%**	**77.6%**
C2_15	6.4	70%	80.6%	80.6%	**82%**	**82%**	**82%**
C2_20	9	71.6%	82%	82%	**85%**	**85%**	**85%**
C2_25	12.4	71.6%	80.6%	82%	83.6%	**85%**	**85%**
C2_30	13	71.6%	80.6%	82%	**83.6%**	**83.6%**	**83.6%**
C2_40	15.5	71.6%	80.6%	82%	**83.6%**	**83.6%**	**83.6%**

The table shows percentage of accuracy from various predictions for 67 known regulatory SNPs. Top10, 15, 20, 25, 30, and 40 represent the accuracy of predicted TF binding affinity changes due to SNPs in the top 10, 15, 20, 25, 30, and 40 of predicted TFs, respectively. Elapsed_time means the CPU times that were used in the prediction. The PWMs_full and C1_full indicate predictions based on 1772 human TFs and clustered PWMs level 1 (e.g., ∼736 pseudo-human TFs), respectively. C2_10, _15, _20, _25, _30, and _40 represent predictions from C2 (clustered PWMs level 2) by selecting the top 10, 15, 20, 25, 30, and 40 TFs from C1, respectively.

### Performance in identifying functional mutations at gene promoters in cancer patient cohort data

Here, whole-genome-sequencing (WGS) data and RNA-seq experiments for tumor-normal paired cancer patient cohorts (e.g., 14 follicular lymphoma samples—FL14, the second cohort of follicular lymphoma, and melanoma or skin cancer patients data) were obtained from ICGC^41^ and earlier publication.^8^ First, a similar pipeline to BayesPI-BAR2^36^ was applied to these datasets by using bpb3 based on 1772 PWMs of human TFs (PWMs_full) to predict the significant mutation blocks (e.g., a minimum of 3 patients and 3 SNPs in a mutation block, a maximum mutation block distance 500bp, and Bonferroni-adjusted p-value < 0.001) in the promoter regions (e.g., +/−1000bp from transcription start site) of protein coding genes with differential expression between tumor and normal samples (T-test of p-value < 0.05). To identify TFs with significant binding affinity changes in the mutation blocks due to DNA sequence variation (e.g., using tumor mutations as background model, multiple chemical potentials (from −8 to −20) for estimating TF binding affinity changes, and Bonferroni-adjusted p-value < 0.05). Then, the same data and parameters were adopted by the new bpb3 function such as using the clustered human TFs (e.g., ∼736 pseudo-human TFs; clustered PWMs level 1—C1_full) to identify TFs with significant binding affinity changes in mutation blocks. Subsequently, the top 15, 20, or 25 pseudo-TFs from C1 foreground calculation were used (e.g., C2_15, _20, or_25), respectively, to predict TFs with significant binding affinities changes caused by DNA sequence variants in the mutation blocks. All calculations were done in the same high-performance computer with 10 parallel processes.

**Figure 2:**
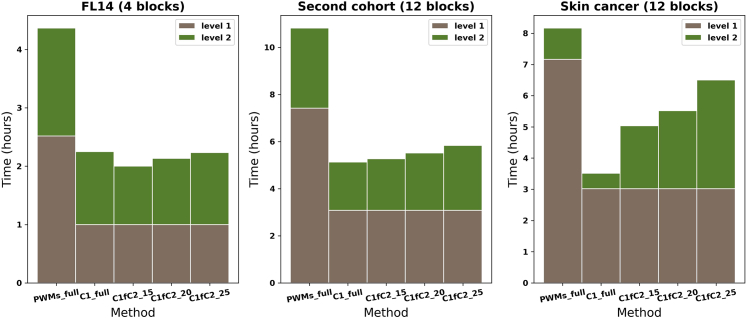
Elapsed time for identifying functional mutation blocks with significant TF binding affinity changes in cancer patient cohorts This figure shows CPU hours used in analyzing WGS and RNA-seq data from cancer patient cohorts by using various combination of the pipeline. All calculations were done in the same computer with 10 parallel processes. FL14 are data from published 14 follicular lymphoma samples, which detected 4 significant mutation blocks (median of mutation block size is 457bp). The second cohort is an independent follicular lymphoma patient (22 patients) cohort download from ICGC, which identified 12 significant mutation blocks (median of mutation block size is 475bp). Skin cancer is a melanoma cancer patient (263 patients) cohort downloaded from ICGC, which predicted 12 significant mutation blocks (median of mutation block size is 144bp). The PWMs_full and C1_full indicate predictions based on the full 1772 human TFs and the clustered PWMs level 1 (e.g., C1 with ∼736 pseudo-human TFs), respectively. C1fC2_15, _20, and _25 represent predictions based on C1 foreground and C2 (clustered PWMs level 2) by using the top 15, 20, and 25 pseudo-TFs from C1, respectively. For PWMs_full and C1_full, the level 1 and the level 2 represent foreground and background calculations, respectively. For C1fC2, the level 1 and the level 2 represent C1 foreground calculation and the selected top N pseudo-TFs in C2 calculation, respectively.

In this analysis, 4, 12, and 12 significant mutation blocks are predicted in FL14, second FL cohort and skin cancer data, respectively. Elapsed CPU times in five different calculations are recorded in Table S1 and Figure 2. For the two follicular lymphoma datasets, almost twice more CPU hours are needed in the original BayesPI-BAR2 with 1772 PWMs (PWMs_full) than that in the bpb3 with either C1_full or a combination of C1 foreground (e.g., ∼736 pseudo-human TFs) with C2 (e.g., the top 15, 20, 25 pseudo-TFs from C1). In these two examples, the cost of the CPU time in the second level calculation (C2) is almost not affected by the number of top TFs selected from the first level (C1 foreground). For the skin cancer data, though C1_full used less than half of CPU time that was needed for a full calculation (PWMs_full), the total CPU time needed for a combination of C1 foreground with the second level calculation (e.g., C2_15, C2_20, C2_25 in Figure S1) is increased from ∼60% to ∼80% of PWMs_full. This is expected as shown in the previous evaluation of 67 known regulatory SNPs (Figure S1). More top TFs from C1_full are included in the second level calculation (C2), hence more CPU time is needed. Nevertheless, there is a plateau for increasing the prediction accuracy by using more top-ranked TFs for C2 (e.g., > =top 20; Figure S1). Therefore, with similar prediction accuracy, combining C1 foreground with its top 20 ranked TFs for C2 calculation may reduce ∼30%–∼50% CPU hours compared to the full calculation (PWMs_full). Please refer to Figure 2 and Table S1 for a detailed illustration of these performance comparisons.

### Evaluation of predicted mutation blocks between the 1772 PWMs and the clustered PWMs

**Figure 3:**
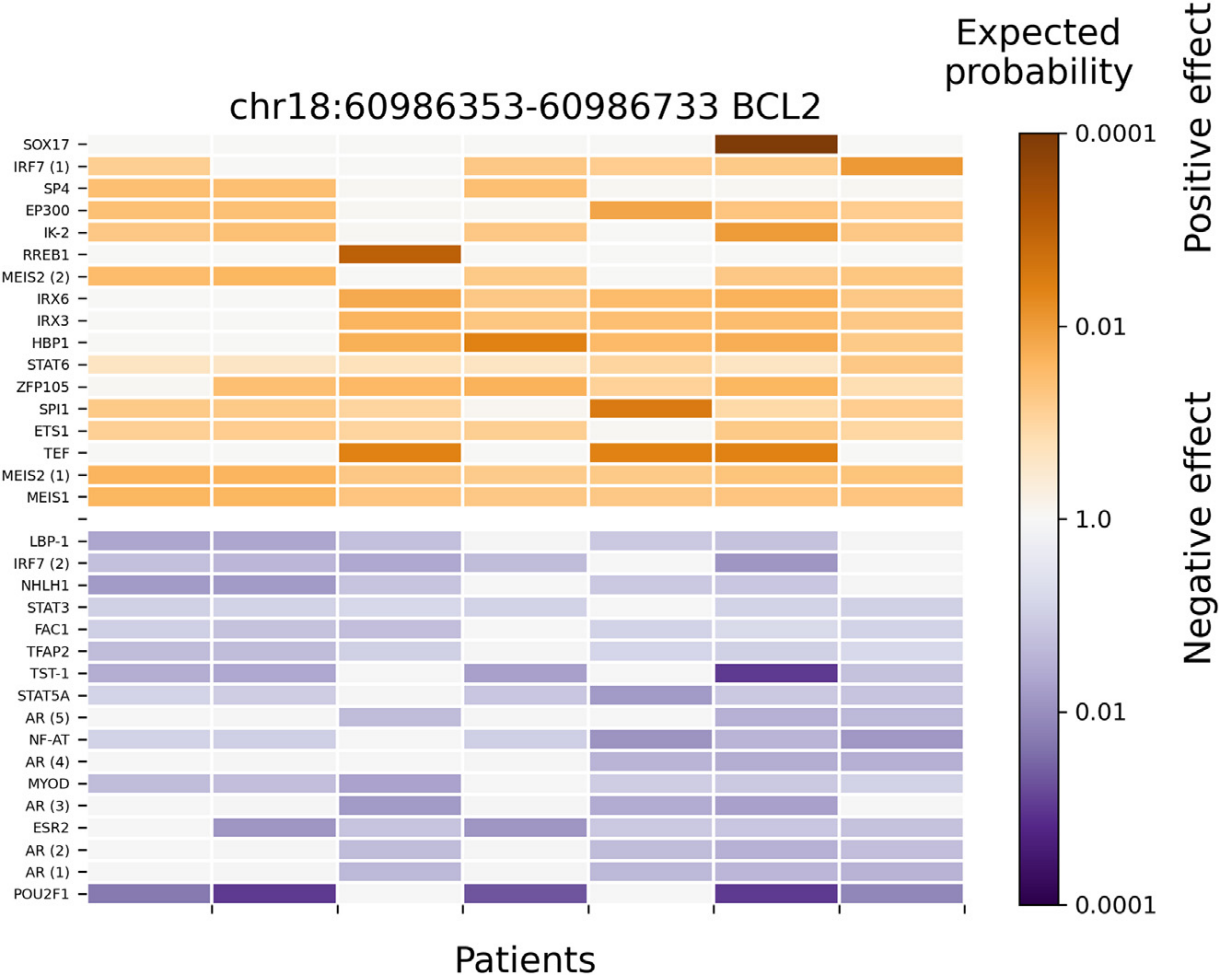
Significant TF binding affinity changes (by PWMs_full method) in the promoter region of *BCL2* where a patient-specific mutation block is predicted from 14 follicular lymphoma patients Around 1772 no-clustered human TF PWMs were used (PWMs_full method) to evaluate their significant binding affinity changes in mutation blocks detected at promoter regions (e.g., +/−1000bp of TSS). The row labels are the TF names, and the column labels are patients with a regulatory mutation block (chr18:60986353-60986733) at the promoter region of *BCL2*. TF names are repeated when several alternative PWMs for a single TF are significantly affected. The color encodes the expected probability that the TF will be affected by random mutations as strongly as by the patient mutations (median size of significant blocks is 457bp), on the logarithmic scale. The positive and negative affinity changes are colored orange and blue, respectively. TFs with very low expression (RPKM < 0.03) were filtered out. Only TFs with significant changes (p < 0.01) across all patients are shown.

**Figure 4:**
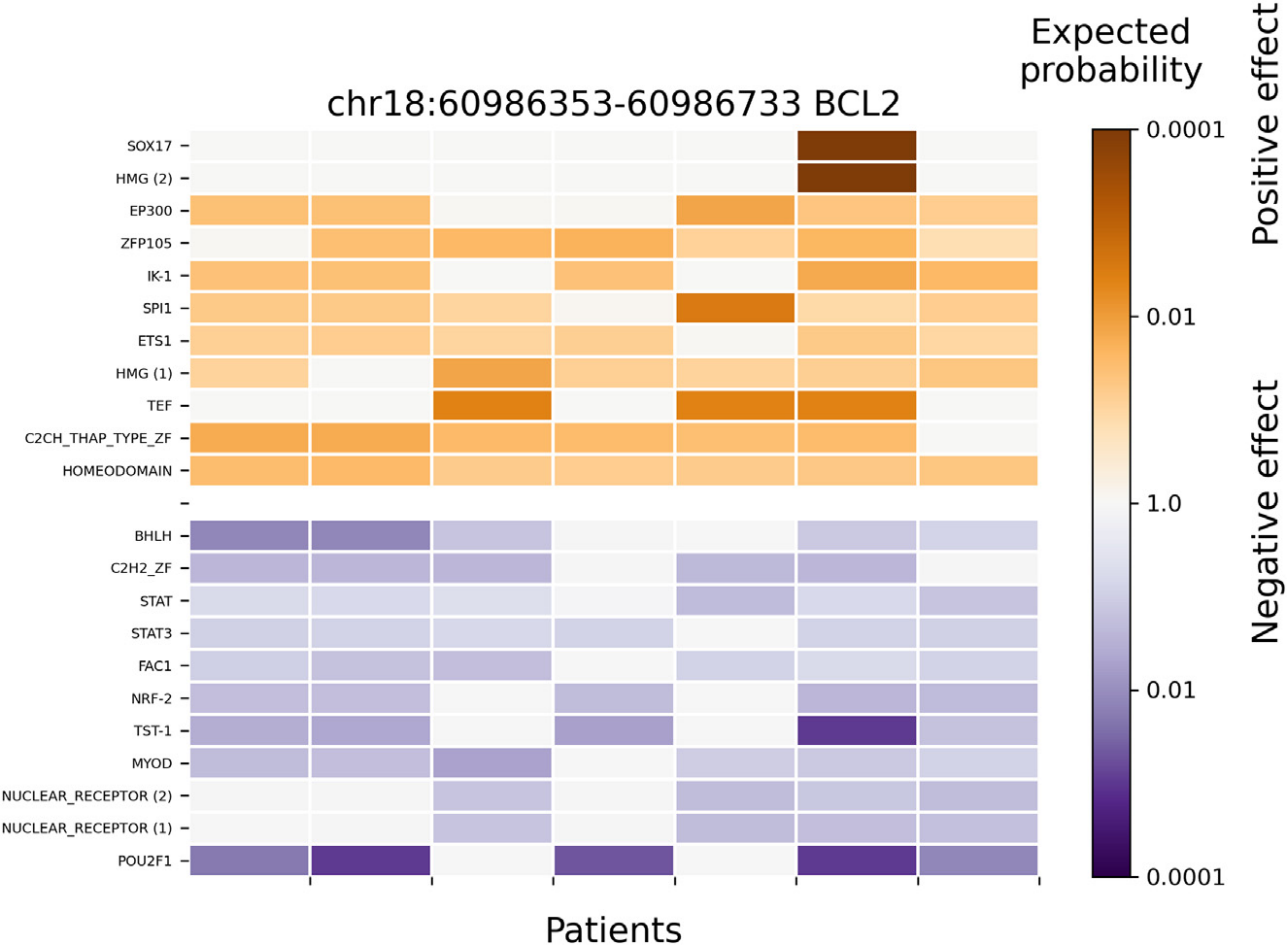
Significant clustered TFs binding affinity changes (by C1_full method) in the promoter region of *BCL2* where a patient-specific mutation block is predicted from 14 follicular lymphoma patients Around 736 pseudo-human (or clustered/representative) TFs PWMs were used (C1_full method) to evaluate their significant binding affinity changes in mutation blocks detected at promoter regions (e.g., +/−1000bp of TSS). The row labels are the TF or DBD name, and the column labels are patients with a regulatory mutation block (chr18:60986353-60986733) at promoter region of *BCL2*. TF or DBD names are repeated when several alternative PWMs for a single TF or DBD are significantly affected. The color encodes the expected probability that the TF will be affected by random mutations as strongly as by the patient mutations (median size of significant blocks is 457bp), on the logarithmic scale. The positive and negative affinity changes are colored orange and blue, respectively. TFs with very low expression (RPKM < 0.03) were filtered out. Only TFs with significant changes (p < 0.01) across all patients are shown. Here, Homeodomain factors include MEIS1 and MEIS2, and the nuclear receptor factor contains AR.

After comparing the accuracy and performance between the original method and the clustered PWMs method in 67 known regulatory SNPs and cancer cohort data, a more detailed evaluation of results between the methods will be illustrated here. For FL14 data, it is known that TFs bindings from nuclear receptors are affected by a mutation block in the promoter of *BCL2* (B cell lymphoma 2).^8^ This known-mutation block (chr18:60986353-60986733) and the corresponding significant TF binding affinity changes were predicted by all five types of calculations (PWMs_full, C1_full, C1fC2_15, C1fC2_20, and C1fC2_25). The heatmaps of significant TF binding changes (p-value < 0.01) at this mutation block from the three predictions are illustrated in Figures 3, 4, and S2 for PWMs_full, C1_full, and for a two-level approach by combining of C1_full and C2 method together (C1fC2_25), respectively. In Figure 3, the result based on 1772 human PWMs (PWMs_full) shows an increasing TF binding affinity for both MEIS1 and MEIS2, but a decreasing one for AR and NHLH1. In Figure 4, a result from clustered PWMs (e.g., 736 pseudo-TFs; C1_full) indicates a positive and negative TF binding effect on the Homeodomain and nuclear receptor factors, respectively. Homeodomain factors include MEIS1 and MEIS2, and the nuclear receptor factor contains AR. In Figure S2, a result of the second level calculation based on the top 25 ranked pseudo-TFs from C1_full (C1fC2_25) reveals a similar result as that shown in Figure 3, the TF binding affinities for both MEIS1 and MEIS2 are positively affected but that for AR and NHLH1 are negatively affected. Thus, the results from clustered PWMs (e.g., C1_full and C1fC2_25) are consistent with the original calculation using 1772 human PWMs (PWMs_full). Importantly, both methods predict that the binding affinity of nuclear receptor is negatively affected by the mutation block in the promoter of *BCL2* gene.

**Figure 5:**
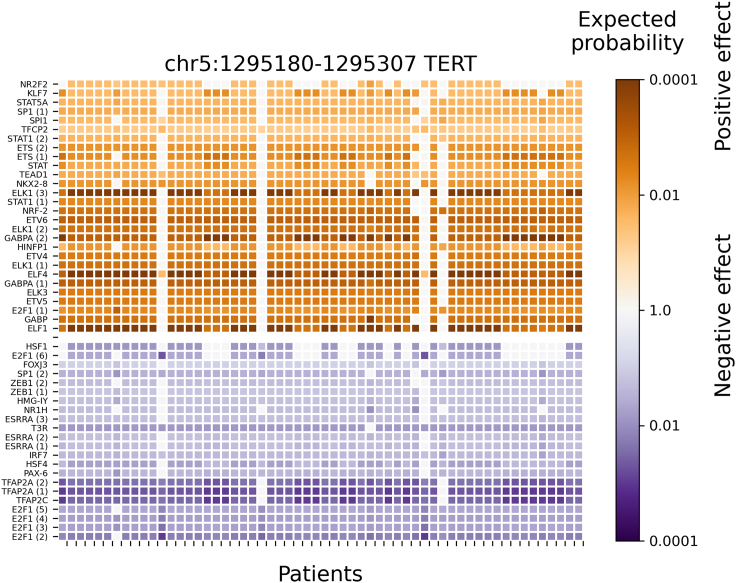
Significant TF binding affinity changes (by PWMs_full method) in the promoter region of *TERT* where a patient-specific mutation block is predicted from 263 melanoma patients Around 1772 no-clustered human TF PWMs were used (PWMs_full method) to evaluate their significant binding affinity changes in mutation blocks detected at the promoter regions (e.g., +/−1000bp of TSS). The row labels are the TF names, and the column labels are patients with a regulatory mutation block (chr5:1295180-1295307) at the promoter region of *TERT*. TF names are repeated when several alternative PWMs for a single TF are significantly affected. The color encodes the expected probability that the TF will be affected by random mutations as strongly as by the patient mutations (median size of significant blocks is 144bp), on the logarithmic scale. The positive and negative affinity changes are colored orange and blue, respectively. TFs with very low expression (RPKM < 0.03) were filtered out. Only TFs with significant changes (Bonferroni-adjusted p value < 0.001) across all patients are shown.

**Figure 6:**
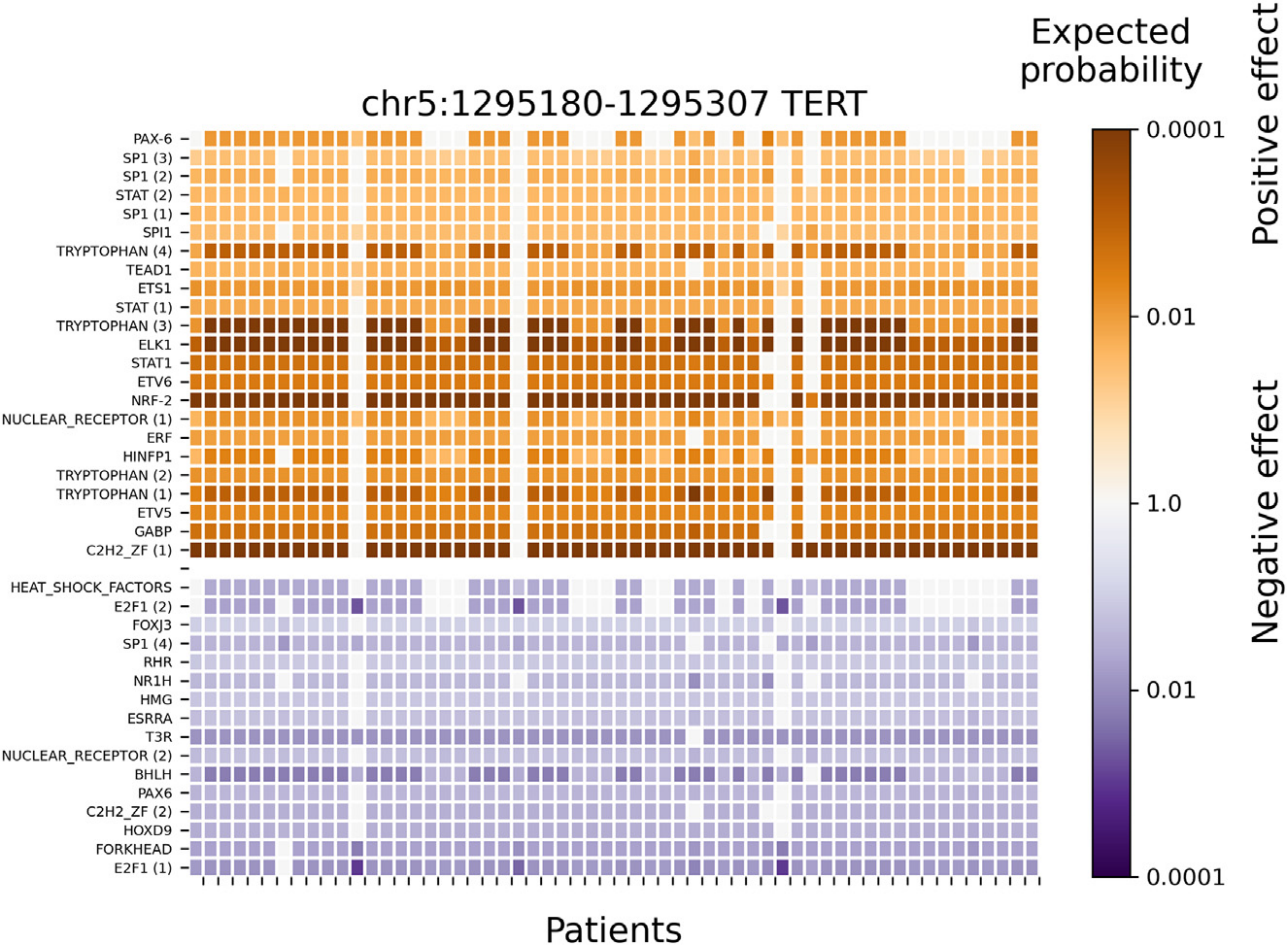
Significant clustered TFs binding affinity changes (by C1_full method) in the promoter region of *TERT* where a patient-specific mutation block is predicted from 263 melanoma patients Around 736 pseudo-human (or clustered/representative) TFs PWMs were used (C1_full method) to evaluate their significant binding affinity changes in mutation blocks detected at the promoter regions (e.g., +/−1000bp of TSS). The row labels are the TF or DBD name, and the column labels are patients with a regulatory mutation block (chr5:1295180-1295307) at promoter region of *TERT*. TF or DBD names are repeated when several alternative PWMs for a single TF or DBD are significantly affected. The color encodes the expected probability that the TF will be affected by random mutations as strongly as by the patient mutations (median size of significant blocks is 457bp), on the logarithmic scale. The positive and negative affinity changes are colored orange and blue, respectively. TFs with very low expression (RPKM < 0.03) were filtered out. Only TFs with significant changes (Bonferroni-adjusted p value < 0.001) across all patients are shown. Here, a Tryptophan includes ETS-related family, and Forkhead contains FOX family.

To further evaluate the results in skin cancer cohort data, the predicted significant TF binding affinity changes at a mutation block (chr5:1295180-1295307; near the promoter of *TERT* gene) are displayed in Figures 5, 6, and S3 for PWMs_full, C1_full, and C1fC2_25, respectively. This mutation block is the most affected by SNPs from the skin cancer cohort (e.g., SNPs from 58 out of 263 patients are located in this mutation block), which verifies the known information that there is a frequent mutation at the promoter of the *TERT* (telomerase reverse transcriptase) gene in melanoma cancer patients.^42^ In Figure 5, a result based on 1772 human PWMs (PWMs_full) indicates that 15 of 28 significantly affected TFs are ETS-related (or Tryptophan cluster factors) with significant positive binding affinity changes (increased TF binding affinity due to mutation) at the mutation block (e.g., ETS, ELK1, ELK3, ELF1, ELF4, ETV4, ETV5, ETV6, SPI1, and GABPA). In Figure 6, a result from clustered PWMs (C1_full) shows that 11 of 23 pseudo-TFs (e.g., SPI1, Tryptophan, ELK1, ERF, ETV6, ETV5, and GABP) belong to an E26 transformation-specific (ETS) related cluster (or Tryptophan cluster). In Figure S3, a result based on TFs in the top 25 pseudo-TFs from the C1 foreground calculation (C1fC2_25) provides TFs with significant binding affinity changes at the block, where 20 out of 29 TFs are ETS-related (e.g., SPI1, ELK1, ELK3, ELK4, ETS1, ETV6, GABPA, FLI1, ETV3, ETV1, ETV4, ETV5, and ELF1). Thus, the conclusions of three predictions (PWMs_full, C1_full, C1fC2_25) are the same: ETS-related factors significantly increased their binding affinity at the mutation block near the promoter of the *TERT* gene in the melanoma cohort. This result is in agreement with a previous publication^42^ that mutations at the promoter of the *TERT* gene generate *de novo* binding motifs for ETS-related factors in skin cancer.

### Comparing prediction accuracies between bpb3 and other methods (BayesPI-BAR, sTRAP and is-rSNP) in 67 verified SNPs

The accuracy of bpb3 was compared with that of BayesPI-BAR^43^ and other tools (e.g., sTRAP^13^ and is-rSNP^12^), on predicting the TF binding affinity changes due to mutations. The sTRAP program was obtained from the previous publication, and the same PWMs used by bpb3 were inputted to the program. For is-rSNP, the prediction results of the same 67 verified mutations were obtained from an earlier study,^39^ where a combination of PWMs from both TRANSFAC^44^ and JASPAR^45^ were used. All methods export a list of ranked PWMs where the binding affinities are significantly altered by given SNPs. Based on the prediction accuracy at 67 verified SNPs, a cumulative accuracy plot of the results obtained by the four programs (bpb3, BayesPI-BAR, sTRAP, and is-rSNP) is illustrated in Figure S4A. It reveals that both bpb3 and BayesPI-BAR reach maximum cumulative accuracies between 81% and 88%, but the sTRAP and is-rSNP give maximum accuracies between 71% and 76%, when the top 50 ranked TFs are considered. However, the cumulative accuracies of bpb3, BayesPI-BAR, sTRAP, and is-rSNP are ∼75%, 70%, 61%, and 51%, respectively, when only considering the top 10 predicted TFs. For the 67 verified SNPs, the rankings predicted by different methods (e.g., bpb3, sTRAP. and is-sSNP) were compared in scatterplots Figure S4B and S4C. The result shows that bpb3 gives better TF rankings than sTRAP and is-rSNP for 51% and 57% of SNPs, respectively. However, only 34% and 33% of SNPs were ranked better by sTRAP and is-rSNP, respectively. These differences are statistically significant based on Wilcoxon signed rank test (p-value < 0.03 and < 0.005 for sTRAP and is-rSNP, respectively). In summary, bpb3 has a similar prediction accuracy as BayesPI-BAR, but gives a better prediction accuracy than the other two tools, based on the evaluation of the 67 verified SNPs.

### Classifying functional regulatory mutations from random mutations in small and large set of SNPs

**Figure 7:**
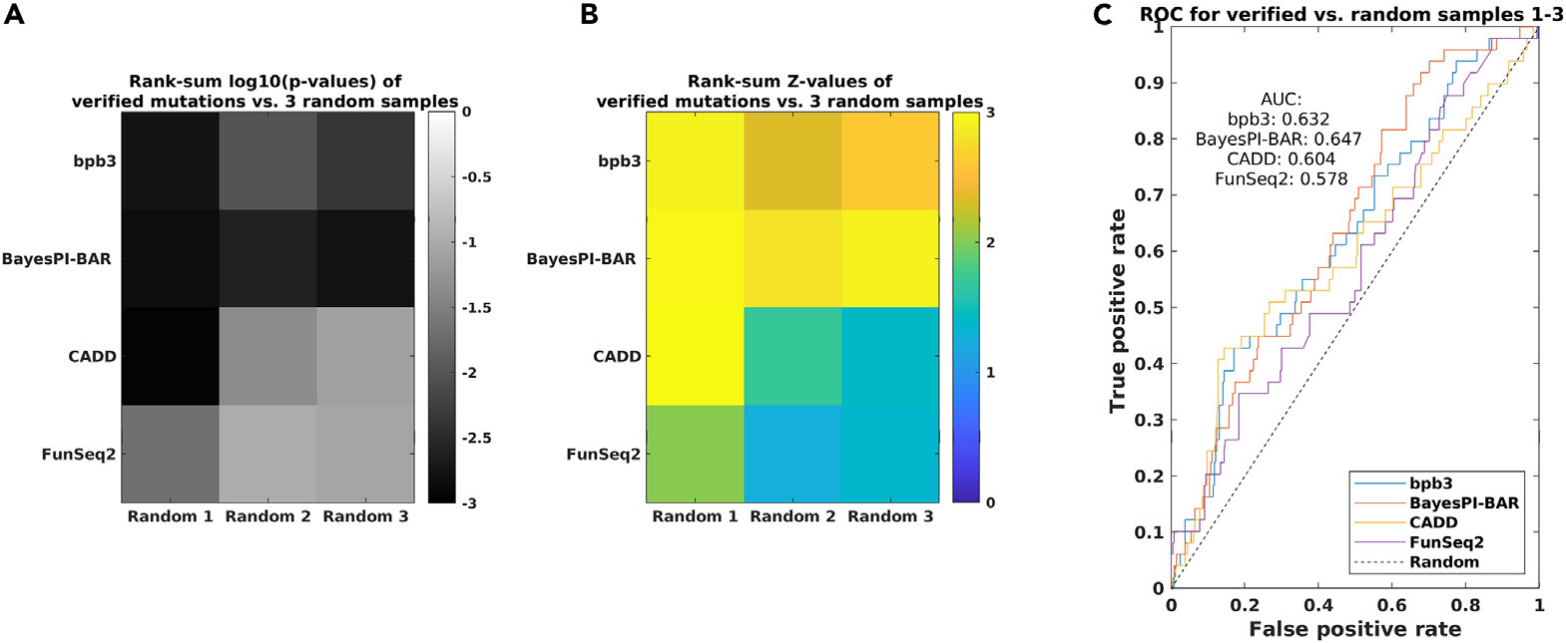
Classifying verified regulatory mutations from random mutations - 67 verified SNPs The classification results of four tools (e.g., bpb3, BayesPI-BAR, CADD, and FunSeq2) are evaluated together based on the 67 verified functional regulatory mutations.^39^ (A) The log10-transformed p-values of the rank-sum tests for the scores of two groups (e.g., verified mutations versus randomly generated ones). Lower p-values indicate a more significant difference of scores between the two groups. (B) The Z-values of the same rank-sum tests as in Figure 7A. The more the yellow, the higher the Z-value. (C) The receiver operating characteristic (ROC) curves illustrate performance comparison of binary classification (e.g., functional versus random mutations) based on the scores generated from four methods (bpb3, BayesPI-BAR, CADD, and FunSeq2). The area under this curve (AUC) represents the degree of separability for the classification. The higher the AUC, the better the method is at classifying two groups. For a random-guessing classifier (“Random”), the AUC of the theoretical ROC curve is 0.5.

To study the accuracy of bpb3 in distinguishing regulatory mutations from random ones, we first used a small set of 67 verified SNPs as functional regulatory mutations and made three randomly generated regulatory mutations (e.g., ∼120 SNPs located at +/−500bp to the TSS of randomly selected protein coding genes) for the evaluation, where mutation scores for the verified mutations and random ones were obtained from the four tools; bpb3, BayesPI-BAR, CADD,^29^ and FunSeq2.^23^ These scores of verified mutations and random ones were compared by Wilcoxon rank-sum tests, and the log10-transformed p-values and the corresponding Z-values are displayed in heatmaps Figures 7A and 7B, respectively. Since some of verified mutations were removed by either FunSeq2 or CADD due to their internal filtering criteria, the results in Figure 7 are based only on 49 verified regulatory mutations and the three sets of randomly generated ones (e.g., each with ∼100 mutations) that passed filtering in both FunSeq2 and CADD as noncoding mutations. In Figure 7A, the darker the color is, the more significant the p-value is, which indicates that mutation scores provided by both bpb3 and BayesPI-BAR reveal a significant difference between the verified and the random mutations (e.g., median p-value < 0.0045 and < 0.002, respectively), but the corresponding scores from either CADD or FunSeq2 only suggest a marginal or weak difference between them (e.g., median p-value < 0.05 or < 0.09). In Figure 7C, the ROC curves (the true positive rate versus the false positive rate) illustrate the accuracy of binary-classification for functional versus random mutations based on the mutation scores obtained from four tools, where the accuracy is slightly higher by bpb3 and BayesPI-BAR than by the other tools. The AUC values for bpb3, BayesPI-BAR, CADD, and FunSeq2 are 0.63, 0.64, 0.6, and 0.57, respectively.

**Figure 8:**
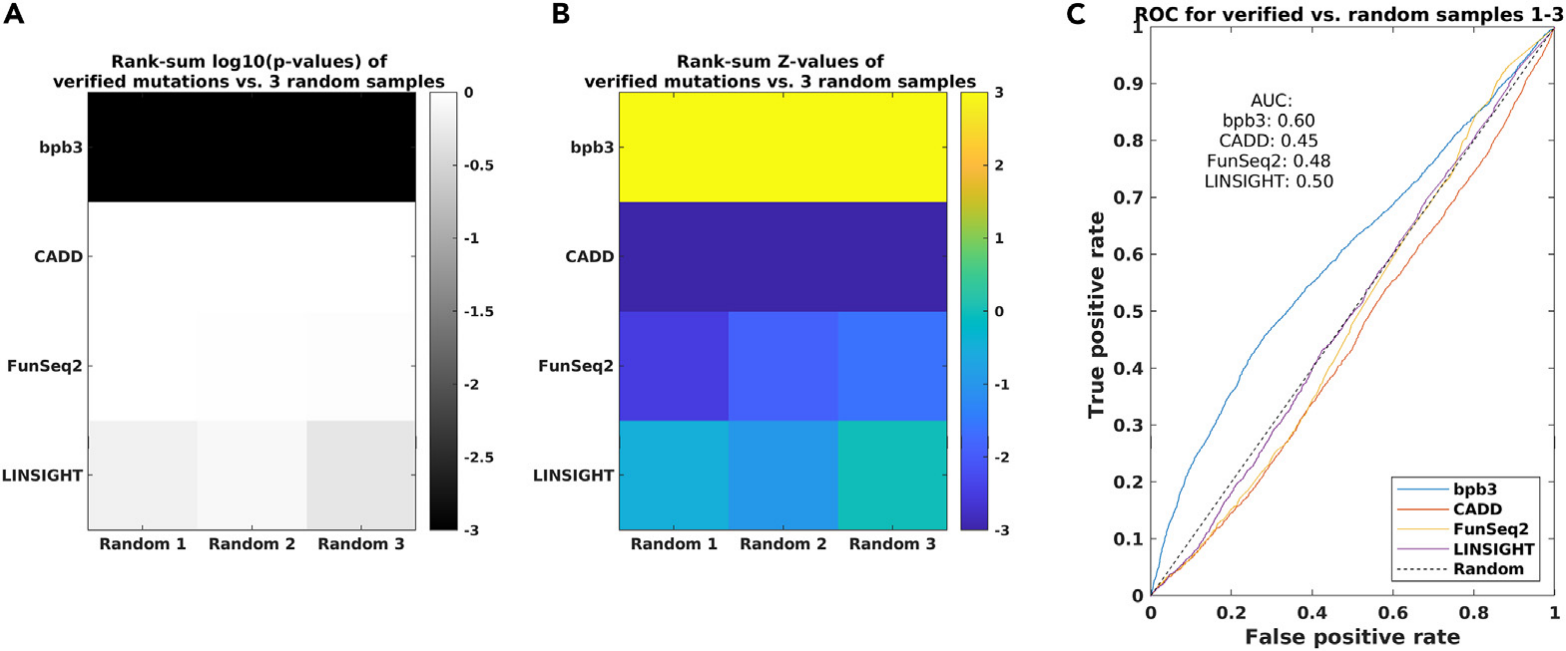
Classifying verified functional regulatory mutations from random mutations – 3075 verified regulatory mutations The classification results of four tools (e.g., bpb3, CADD, FunSeq2, LINSIGHT) are evaluated together based on the 3075 verified functional regulatory mutations.^46^ (A) The log10-transformed p-values of the rank-sum tests for the scores of two groups (e.g., verified mutations versus randomly generated ones). Lower p-values indicate a more significant difference of scores between the two groups. (B) The Z-values of the same rank-sum tests as in Figure 8A. The more the yellow, the higher the Z-value. (C) The receiver operating characteristic (ROC) curves illustrate performance comparison of binary classification (e.g., functional versus random mutations) based on the scores generated from four tools (bpb3, CADD, FunSeq2, and LINSIGHT). The area under this curve (AUC) represents the degree of separability for the classification. The higher the AUC, the better the method is at classifying two groups. For a random-guessing classifier (“Random”), the AUC of the theoretical ROC curve is 0.5.

Secondly, we performed the same analysis on a large number of verified regulatory mutations (∼3075 SNPs) based on massively parallel reporter assays (MPRAs) with three sets of randomly selected nonfunctional ones (∼3075 SNPs in each set) from a previous study,^46^ by using four different tools (bpb3, CADD, FunSeq2, and LINSIGHT). In Figure 8, the results are based on ∼2915 functional regulatory mutations and the three sets of nonfunctional ones (e.g., each with ∼2910 mutations) that passed filtering in both FunSeq2 and CADD as noncoding mutations. It indicates that the mutation scores generated by bpb3 show significant difference between the verified and the random one (e.g., median p-value of rank-sum test < 0.001; Figures 8A and 8B), but the scores from the other three tools could not distinguish them (e.g., median p-value of rank-sum test < 1). Particularly, the prediction accuracy of bpb3 is quite stable for a large number of mutations (e.g., AUC value = 0.60; Figure 8C). However, for the rest of tools, the classification of verified mutations and random mutations is poor based on their mutation scores (e.g., Figure 8C; AUC value = 0.45, 0.48, and 0.5 for CADD, FunSeq2, and LINSIGHT, respectively). Thus, a better classification of mutations (e.g., functional versus nonfunctional ones) can be achieved by using mutation scores from bpb3 than that by scores from the other three tools tested.

### Applying bpb3 to identify mutation blocks functionally affecting DNA methylation status and gene expression on predefined genomic regions

A previous publication^35^ reports that the putative regulatory mutation blocks are frequently close to the differential DNA methylation regions (DMRs) at enhancers/promoters by integrated analysis of whole genome sequencing, DNA methylation, gene expression, and topological associating domain information in 14 follicular lymphoma patients. From that study, two significant mutation blocks at chr18 are nearby the promoter of the *BCL2* gene (block_66303∼chr18:60983692_60986805; block_66304∼chr18:60988053_60988779) and overlapping with both DMR and enhancers. In particular, there is a significant FOX family TF binding affinity changes between the FL patients and the normal samples at block_66304, which was verified in lymphoma cancer cell lines previously.^8^ For that reason, we obtain the predicted DMRs that overlap with enhancers in chromosome 18 from the study^35^ and compile them into a list of predefined genomic regions (e.g., including an additional 5kb flank region per side on two sides of each DMR). Subsequently, bpb3 was applied to an independent follicular lymphoma test cohort (22 patients)^8^ based on these predefined genomic regions, to predict the significant mutation blocks and their TF binding affinity changes in FL. Five mutation blocks are identified and three of them are defined as significant mutation blocks by bpb3 (Bonferroni-adjusted p-value < 0.001; Table S2). One of the significant mutation blocks (block_4_18_60987833_60988772) overlaps to a mutation block predicted previously from 14 FL patients (e.g., block_66304∼chr18:60988053_60988779).^35^ This newly identified mutation block (block_4_18_60987833_60988772) also causes significant TF binding affinity changes for the FOX protein family (Figure S5) in the second FL cohort (22 patients). Thus, a search of significant mutation blocks in a set of predefined genomic regions (e.g., DMRs overlapping with enhancers in chr18 from 14 FL patients^35^) reaches the same conclusion as the original study,^8^ when applying bpb3 on an independent FL test cohort (22 patients).

### Predicting mutation block and gene associations based on diverse functional genomic data

To study the potential applications with diverse functional genomic data (e.g., histone modifications, epigenetic DNA methylation, nucleosome density, transcriptome data) to predict putative functional regulatory mutation block-gene associations, we first identified genome-wide mutation blocks (e.g., 786 blocks with at least 2 SNPs and from at least 2 patients) and differentially expressed genes (DEG; ∼5843 genes with T-test p-value < 0.05 between the two groups) from the second FL cohort (∼181135 of SNVs from 22 patients) by using the bpb3 package. Then, the 786 mutation blocks were annotated to four genomic regions (Gene, TSS, TES, and 5’distance region), from which an initial list of mutation block-gene associations was built. Subsequently, the common topologically associating domains (TAD) information in five human cell lines, as well as the differential methylation regions (DMR) between tumor and control samples, were acquired from earlier publications.^35^^,^^47^ These were utilized to filter the initial list of mutation block-gene associations, such that the mutation blocks are either overlapping with DMRs or their associated genes are differentially expressed. In addition, requiring that a pair of mutation block-gene association is located within the same TAD.^35^ Consequently, ∼175 mutation blocks and ∼158 differentially expressed genes (DEGs) passed such filtering.

**Figure 9:**
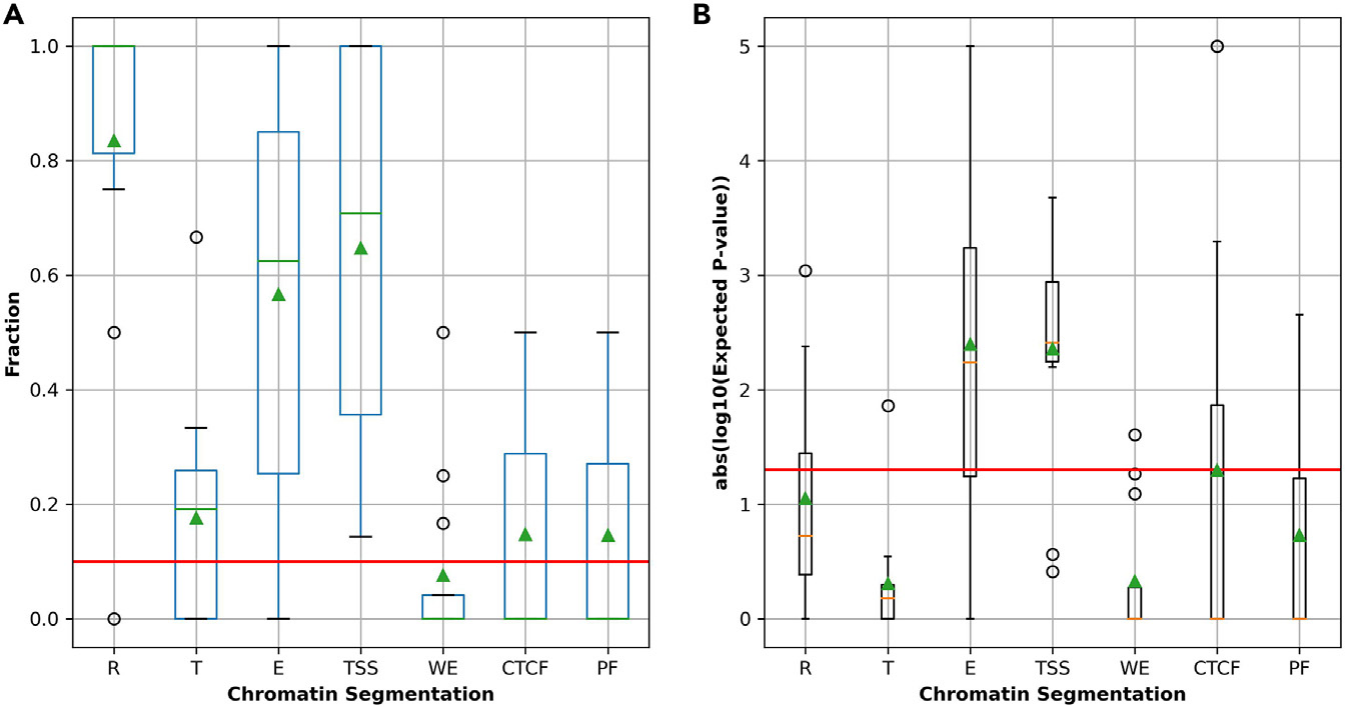
Boxplots of the fraction and the expected p-values of the enrichment test for mutation block-gene associations in seven chromatin states The mutation blocks of block-gene associations are predicted in the second follicular lymphoma (FL) cohort (22 patients), which are enriched (e.g., expected p-value < 0.05) in either TSS or enhancer regions according to the enrichment test in seven chromatin states. (A) Boxplots of the fraction of mutation blocks for a gene that are overlapping with the seven chromatin segmentations (or chromatin states) in human genome, where the red smooth line and the green triangle represent the 10% of mutations blocks and the mean values of a boxplot, respectively. (B) plots the absolute log10 (or -log10) of expected p values for mutation blocks enriched in the seven chromatin segmentations from a permutation test with 10000 randomly selected blocks, where the red smooth line and the green triangle indicate an -log10 of expected p value = 1.3 (or expected p value = 0.05) and the mean values of a boxplot, respectively. Seven chromatin states are R, T, E, TSS, WE, CTCF, and PF which indicate the predicted repressed/low activity region, transcribed region, enhancer, promoter region/transcription start site, weak enhancer/open chromatin region, CTCF enriched element, and promoter flanking region, respectively.

Finally, a random permutation test was used to evaluate the enrichment of mutation blocks associated with genes across seven chromatin states. These seven chromatin states represent functional regions in the human genome (e.g., TSS, PF—promoter flank region, E—enhancer, WE—weak enhancer, CTCF, R—repression region, and T—transcribed region), which were predicted previously by applying ML methods on multiple functional genomic datasets (e.g., histone modifications—H3K4me1, HeK4me2, H3K3me3, H3K9ac, H3K27ac etc., and nucleosome density) across six human cell-lines.^47^^,^^48^ In the end, the mutation blocks associated with a gene that are highly enriched in either TSS or Enhancer^35^ (e.g., an expected p-value < 0.05 based on 10000 times random permutation test) are selected as putative FMB-gene associations: ∼51 mutation blocks with 12 genes predicted by the enrichment test in seven chromatin states (Table S3 for detailed description). Figure 9A shows most of mutation blocks associated with 12 putative target genes are overlapping with R (repressed regions > 80%), TSS (> 60%), and E (enhancer > 60%) regions, and are significantly enriched in two chromatin states (TSS and Enhancer; the mean of expected p-values < 0.01). Particularly, two (*BCL2* and *BCL6*) of twelve genes were known previously^8^^,^^49^ to be associated with regulatory mutation blocks in FL (e.g., yellow highlighted in Table S3; block_493_18_60987833_60988858, block_492_18_60983828_60987060, and block_631_3_187461042_187463057) based on another independent patient cohort. Thus, diverse functional genomic data not only improves the prediction accuracy for functional regulatory mutation blocks, but also remarkably reduces the number of putative target genes (e.g., from an initial 786 mutation blocks associated with 224 genes, to the final 51 blocks paired with only 12 genes).

## Discussion

Bpb3 can identify functional mutation blocks and predict the effect of these mutations on TF-DNA binding based on whole genome sequencing data from cancer or other disease patients. Compared to other functional SNVs prediction and annotation tools, bpb3 is unique in terms of its advantage in searching for functional regulatory mutation regions by integrating multi-omics data from patients. The integration of transcriptomic and other omics data is one of its specific features. These features will not only improve the predication accuracy for functional mutation blocks, but also help finding the mutation target genes which may be of importance in precision medicine. The functional mutation blocks predicted by bpb3 are based on the following features: (1) carrying high mutation frequency with a cluster of SNVs cross patients; (2) resulting in significant change in protein-DNA binding affinity in a list of top-ranked TFs; (3) targeting in no-coding *cis*-regulation regions such as promoters or enhancers; (4) being nearby or overlapping with DEGs or/and other interesting function-associated regions such as differentially methylated regions (DMR), and thus functionally associating with dysregulation in gene expression. In summary, bpb3 can automatically predict functional mutation blocks by integrating genome, transcriptome and epigenomic data from patients.

The bpb3 package is tested and evaluated on both small and large datasets from cancer patients. Its predicted results for 263 skin cancer of melanoma (MN) and 14 FL patients are accurate and reproducible, by using the three evaluation methods for detecting significant TF binding affinity changes caused by SNPs. The first method (PWMs_full) uses all available PWMs, while the second method only considers clustered PWMs (C1_full). The third one (C1fC2_N) uses a two-level approach to predict significant TF binding affinity changes: the clustered PWMs are used in foreground calculations (first level); the top N clustered PWMs from the first level predictions are extracted and PWMs within the selected clusters are used for further calculations (second level). The results of the three types of calculations match to each other to a large extent. In general, C1_full provides accurate prediction as the other two methods but with far fewer CPU hours. Thus, C1_full is the optimized method in general for big data computation and if the CPU time is one of the main concerns. However, C1_full identified less TFs compared to PWMs_full method, because the number of the reference TFs have decreased after PWMs classification. PWMs_full method is recommended for detailed studies in small datasets, while the C1fC2_N method is suited for narrow bottom-up TFs candidates, hence speeding up computation time.

Remarkably, when the accuracy of the top ranked TFs from bpb3 was compared against the previous BayesPI-BAR^36^ and other similar tools (e.g., sTRAP^13^ and is-rSNP^12^) based on 67 verified SNPs,^39^ the bpb3 gives better true TF ranking than the other tools (Figure S4). In an evaluation of binary classification between the verified 67 SNPs and the randomly selected ones based on mutation scores obtained from different methods (e.g., bpb3, BayesPI-BAR,^36^ CADD,^29^ and FunSeq2^23^), bpb3 has the highest AUC (Figure 7) as compared to the other tested methods. Similarly, bpb3 keeps a better classification than other tools in a big data of ∼2915 verified regulatory mutations from MPRA study^46^ (Figure 8). Additionally, diverse functional genomic datasets (e.g., histone markers in enhancer and promoter regions, nucleosome density, DNA methylation, gene expression, and TAD) were integrated into the second FL cohort (22 patients) for predicting the functional regulation mutation block-gene associations. It results in reducing the number of putative functional regulatory mutation blocks and their associated target genes largely (e.g., from ∼786 to ∼51 blocks, and from ∼224 to ∼12 genes). It not only recovers the known regulatory mutation block-gene associations in FL (e.g., *BCL2* and *BCL6* genes^8^^,^^43^; Table S3), but also reveals the regulatory mutation blocks are highly enriched in TSS and enhancer regions (e.g., expected p-value < 0.01; Figure 9). Thus, it is a great advantage to integrate diverse functional genomic datasets (e.g., histone modifications, nucleosome density, DNA methylation, and TAD) in a functional regulatory mutation block study. Integration of such additional omics data will be implemented in future bpb3 package.

Recently, a sequence classes-based tool Sei, an improved version of DeepSEA Beluga, has been developed to classify and quantify (score) the regulatory impact of SNVs on DNA sequences.^50^ The whole genome sequences are tiled into million fragments (each at about 4 kb) and classified by deep learning. The training data are collected from the Encyclopedia of DNA Elements (ENCODE) and Roadmap Epigenomics projects, which include the information of histone marks, TFs binding and chromatin accessibility profile such as DNase I sensitivity. This model could accurately predict the effect of individual SNVs on TF binding. The advantage is its ability to integrate information from multiple-omics, especially a number of epigenome information. It is robust and efficient for searching on short sequences in web applications. However, this method cannot be applied to integrate patient-specific omics data directly (e.g., RNA-seq, WGS data or epigenetic data) to predict functional mutation blocks and the corresponding impact on TF binding. It is possible to perform both Sei and bpb3 analysis separately, then to filter the top-ranked functionally mutation blocks by SNVs scores from Sei analysis. The FABIAN is another web-interface SNVs tool, which predicts TF binding affinity change on SNVs by combining the information of both PWMs and the transcription factor flexible model (TFFMs). TFFMs are based on hidden Markov models by considering complex positional dependence of PWMs, which may provide more accurate predictions than PWMs.^51^ Thus, to improve the accuracy of predicting TF-DNA binding affinity change, TFFMs might be integrated into bpb3 in the future, or bpb3 can be applied together with other SNVs analysis tools (e.g., Sei and FABIAN) for identifying functional regulatory mutations in cancer or disease by using patient-specific sequencing data.

Bpb3 is a command line tool, which not only includes all R language functions of previous BayesPI-BAR^39^ and Python2 modules of BayesPI-BAR2,^17^ but also adds several new functions and modules in the package (e.g., preprocess of download data from ICGC data portal, generate a heatmap for predicted significant TF binding affinity changes, estimate significant TF binding affinity changes by using either clustered PWMs or a two levels approach, and clean temporary files). Especially, the previous BayesPI-BAR programs were used on various command line scripts, which may not be suited for users with limited knowledge of Python. The Bpb3 is upgraded to Python3 and can be easily installed in either Linux or MAC OS system, in which users can simply call it like other build-in commands in the operating system. In particularly, the bpb3 provides a precompiled input parameter configure file, where the user can easily modify various parameters associated with running of the pipeline. Then, the parameter configuration file can be directly loaded into the bpb3 package for running the analysis automatically, which significantly simplifies the application of bpb3 in different tasks. It is recommended to use high-performance-computing with good size memory because the parallel computation allows the user to run bpb3 efficiently. A brief description of the high-performance-computer that was used in the current study, and the basic steps for installing and applying bpb3 on a similar system (e.g., cloud machine), as well as the usage of CPU hours and memory for bpb3 are provided in the Table S4 and bpb3 online documentation (https://bpb3.github.io/bpb3/). This information may help users to estimate the cost of running bpb3 on a cloud machine based on commercial providers.

In the current cancer cohort studies, bpb3 correctly predicted significant TF binding affinity changes at mutation blocks near the promoter of *TERT* and *BCL2* genes in MN and FL, respectively. It is applicable to genome sequence variants and gene expression data of any other diseases, to identify functional non-coding SNVs in whole genome data and provide reliable predictions of functional mutation blocks affecting TFs binding.

### Limitations of the study

A limitation of current study is that only two crucial features (transcription factor binding affinity changes and differential gene expression) are considered in identifying functional regulatory mutation blocks. While these features are important in gene regulation, other relevant features, such as histone modification marker, DNA methylation patterns, nucleosome density, and Topologically Associating Domains (TADs), were not included in the analysis. Therefore, some functional regulatory mutation blocks may be missed in the prediction. In the future, a comprehensive analysis method shall be developed by incorporating diverse information (e.g., epigenome and TAD), which will improve the understanding of the complex regulatory landscape affected by mutations.

## STAR★Methods

### Key resources table

**Table:** 

REAGENT or RESOURCE	SOURCE	IDENTIFIER
**Software and algorithms**		

Bpb3 (Python package)	This paper	https://bpb3.github.io/bpb3
BayesPI-BAR (R scripts)	(Wang and Batmanov, 2015)^39^	https://junbaiw.github.io/BayesPI-BAR/
BayesPI-BAR2 (Python package)	(Batmanov et al., 2019)^8^	https://junbaiw.github.io/BayesPI-BAR2/
Abc4pwm (Python package)	(Ali et al., 2022)^40^	https://github.com/abc4pwm/abc4pwm
HMST-Seq-Analyzer (Python package)	(Farooq et al., 2020)^52^	https://hmst-seq.github.io/hmst/
STRAP (C++ package)	(Manke et al., 2010)^13^	http://trap.molgen.mpg.de/cgi-bin/home.cgi
CADD (Web tool)	(Rentzsch et al., 2019)^29^	https://cadd.gs.washington.edu/
FunSeq2 (Web tool)	(Fu et al., 2014)^23^	http://funseq2.gersteinlab.org
LINSIGHT (C++ package)	(Huang et al., 2017)^31^	https://github.com/CshlSiepelLab/LINSIGHT

### Resource availability

#### Lead contact

Further information and requests for resources should be directed to and will be fulfilled by the lead contact, Junbai Wang (junbai.wang@medisin.uio.no).

#### Materials availability

This study did not generate new unique reagents.

### Method details

#### Data sources for this study

Here, 67 SNVs with experimentally verified effects of human TF binding (20 from,^53^ 18 from,^1^^,^^37^ and 29 from HGMD database^38^), 3075 verified regulatory mutations and three sets of randomly selected noncoding mutations based on massively parallel reporter assay (MPRA), 1772 human TFs position weight matrix (PWMs), whole-genome-sequencing data (WGS), and RNA-seq experiments for tumor-normal paired cancer patient cohorts^43^^,^^54^ are obtained from previous publications^8^^,^^36^^,^^39^^,^^46^ and International Cancer Genome Consortium^55^ (ICGC), respectively. The 14 follicular lymphoma (FL) patients,^56^ the second cohort of follicular lymphoma with 22 patients,^57^ the 263 melanoma cancer or skin cancer patients (e.g., MELA-AU, SKCA-BR, and SKCM-US projects), and the 776 breast cancer patients (e.g., BRCA-EU, BRCA-KR, BRCA-US, BRCA-FR, and BRCA-UK projects) were obtained from ICGC data portal, respectively. Human TF DNA binding domain (DBD) information is retrieved from three sources (TFClass,^58^ Human Transcription Factors,^59^ and JASPAR^60^). The clustering of 1772 PWMs using DBD information was achieved by the abc4pwm package.^40^ Human enhancer annotation of 197 tissues/cells were obtained from the EnhancerAtlas2.0 database^61^ in the hg19 genome. To define promoter regions (e.g., +10kb/-10kb to transcription start site -TSS) and 5′distance up/down regions (e.g., from 1Mb to 10kb upstream/downstream of the TSS), gene annotation from the GENCODE^62^ (v19 for GRCh37) was used by HMST-Seq-Analyzer.^52^

#### Biophysical theory for BayesPI-BAR

A Bayesian method^63^ for protein–DNA interaction with binding affinity ranking (BayesPI-BAR) was developed previously,^39^ which can be used to quantify the effect of sequence variations on protein binding. For example, to predict putative target transcription factors (TFs) whose binding affinity is significantly affected by the mutation of DNA sequences, or to rank the significance of TF binding affinity changes due to the mutation (e.g., nucleotide polymorphisms -SNPs) at regulatory regions. BayesPI-BAR includes two major parameters (TF chemical potentials or protein concentrations and differential TF binding affinity) that are based on a biophysical theory of protein-DNA interactions,^64^ and the binding probability between the protein and the DNA sequences is calculated by a Fermi–Dirac formula,

$$P\left(S\right)=1/\left(1+e^{\left(E\left(S\right)-\mu \right)/k*T}\right)$$

Where *S* represents the DNA sequence to be bound by a protein, *E(S)* is the protein binding free energy in sequence *S* which corresponds to a position-specific affinity matrix (PSAM), or position-weight matrix (PWM)^65^ of a TF, *mu (μ)* is the chemical potential (or protein concentration), *k* is gas constant, and *T* is the absolute temperature. The TF chemical potential *mu (μ)* at various conditions can be estimated by BayesPI2+,^66^ a Bayesian nonlinear regression model that fits a known TF PWM to an *in vivo* ChIP-seq experiment. Nevertheless, the *mu (μ)* can also be manually adjusted for a given PWM^39^ (e.g., by setting a range from 0 to 23 if there is no ChIP-seq data for a TF). The *differential binding affinity* (*dbA*) is used to distinguish between the direct and the indirect protein–DNA interactions,^66^ which can be used to estimate the TF binding affinity changes between the target DNA sequence and the randomly mutated sequence (background sequence). In this way, a *shifted differential binding affinity* (*δdbA*) is designed to calculate the difference in TF binding affinities between the reference sequence (*S*_*i*_, reference) and the mutated DNA sequence (*S*_*i*_, mutated): *δdbA(S*_*i*_*)* = *dbA(S*_*i*_*, reference)* – *dbA(S*_*i*_*, mutated)*. A similar *δdbA(S*_*i*_*)* calculation is applied to a set of randomly mutated DNA sequences to estimate the significance of such TF binding affinity changes. Subsequently, it can rank TFs based on their impact on the TF-DNA binding affinity changes due to a DNA mutation. In other words, a TF with the strongest impact on TF-DNA binding affinity change will have the largest absolute value of *δdbA* due to a regulatory sequence variation. More information of BayesPI-BAR is given at online documentation (https://bpb3.github.io/bpb3/) and published papers.^39^^,^^67^

#### Integration of genome sequencing data with RNA-seq data

In order to predict functional regulatory mutations in cancer or other disease patients as BayesPI-BAR2,^36^ the BPB3 package possesses the ability to integrate both DNA mutation information (e.g., SNPs from WGS) and gene expression profiles (RNA-Seq) of the corresponding disease cohort. This integration reveals the dysregulation of transcription factors (TFs) when the binding to a DNA sequence is significantly affected by sequence variations (e.g., SNPs of functional regulatory mutations) in disease.^8^ First, BayesPI-BAR finds mutation blocks (e.g., with high mutation frequency across patients) at promoter regions of differentially expressed genes - DEGs (e.g., p-value <0.05 of a two-sample Kolmogorov-Smirnov test/T-test between tumor and normal samples). The patient-specific mutation blocks at gene promoter regions are predicted by MuSSD (Mutation filtering based on the Space and Sample Distribution) algorithm.^8^ Then, a collection of PWMs of known human TFs (e.g., ∼1772 PWMs for ∼700 human TFs) is used to predict a list of top-ranked TFs, whose binding affinities are significantly changed due to the patient-specific mutation blocks. An evaluation of the significant TF-DNA binding affinity changes is based on following three major steps.

(1) **Foreground calculatio**n: all predicted patient-specific mutation blocks are considered foreground mutation blocks, which are tested against all available PWMs of human TFs (e.g., ∼1772 PWMs).
(2) **Background calculation:** background mutation blocks are extracted randomly from regions of interest (e.g., promoters) with the same sequence length as patient-specific mutation blocks. Mutations in each background mutation block are generated based on the reference genome by altering the nucleotides in random positions with either tumor-derived mutations or a given k-mer mutation probability distribution (the mutation signature).^36^
(3) **A comparison between the foreground and the background calculation:** for each patient-specific mutation block, a TF with a distribution of *δdbA* values are significantly different (e.g., P-value <0.05 in a Wilcoxon rank-sum test) between the foreground and the background calculations is considered as a putative target for the mutation block.

More information about the computation please refer to bpb3 online documentation (https://bpb3.github.io/bpb3/) and published work.^36^

#### Three computing methods including a two-level approach in bpb3

In the bpb3 package Figure 1, there are three types of calculations to evaluate the significance of TF binding affinity changes caused by DNA sequence variants. First, to assess significance of TF binding affinity changes due to sequence variants by using all available TF PWMs (PWMs_full method), originally implemented in BayesPI-BAR2, which compares between the foreground and the background TF binding affinity changes caused by sequence variants by using all available TF PWMs. Second, to assess the TF binding affinity changes based on clustered PWMs or PWMs of pseudo-TFs (C1_full method), in which both foreground and background TF binding affinity changes are calculated by the representative PWM (or pseudo-TF) of clustered PWMs (Figures S6 and S7). Third, to predict significant TF binding affinity changes by using a two-level approach, where foreground calculation is based on the clustered PWMs (level 1), then the top N clustered PWMs (pseudo-TFs) from the level 1 are extracted, all PWMs clustered in these selected pseudo-TFs are used for a second round (Clustered PWMs level 2, C2 method) calculation - comparison between foreground and background TF binding affinity changes due to sequence variants (C1fC2_N). Though the C1_full method is the fastest method for obtaining a result because of the clustered PWMs (or pseudo-TF) is far less than all TF PWMs, it can only narrow down a result to a specific cluster of PWMs (or a pseudo-TF). Both PWMs_full and C1fC2_N methods can identify the binding of individual TF that may be significantly affected by DNA sequence variations. C1fC2_N requires fewer CPU hours than the PWMs_full, because the two levels filtering in C1fC2_N significantly reduces the number of PWMs needed in evaluation.

#### Evaluation of TF ranking accuracy between bpb3 and other methods

First, we applied a cumulative accuracy plot to illustrate the performance of bpb3, BayesPI-BAR, sTRAP, and is-rSNP, respectively, for finding the true targets from the top ranked TFs of 67 verified mutations. In the figure, the X axis is the rank of TFs which are selected for evaluating the accuracy, and the Y axis is the fraction of mutations that with the true TFs recovered with the selected or lower rank. Then, we compared the ranking (i.e., bpb3 versus sTRAP/is-rSNP) of predicted true TFs for all mutations by using a scatterplot, where circles represent mutations, X axis and Y axis are the rankings of true TFs predicted by bpb3 and sTRAP/is-rSNP, respectively. Given that a lower rank shows a better prediction, mutations above the red diagonal line indicates a better ranking by bpb3. However, mutations below the diagonal line suggests the ranking by sTRAP/is-rSNP is more accurate. A significance of the difference of ranking between the two methods in a scatterplot is tested by a Wilcoxon signed-rank test.

#### Classification of functional regulatory mutations from random mutations

Two datasets are used in this evaluation. First, 67 verified (or functional)^39^ and three sets of randomly generated regulatory mutations (e.g., ∼300 SNPs randomly selected from +/−500 bp to the TSS of protein coding genes) were used to calculate putative functional mutation scores from the four tools (e.g., bpb3, BayesPI-BAR, CADD^29^ and FunSeq2^23^), respectively. Then, a large number of verified functional regulatory mutations (∼3075 SNPs overlapping in two types of cells, downloaded from https://osf.io/pjxm4/wiki/home/) with three sets of randomly selected nonfunctional mutations (∼3075 SNPs in each set) from massively parallel reporter assays (MPRAs),^46^ were used to compare the performance between the bpb3 and the other three tools (e.g., CADD, FunSeq2, and LINSIGHT) based on the same mutation score calculation in the first evaluation. For bpb3 and BayesPI-BAR, the mean absolute scores of δdbA values^39^ of the top 20 predicted TFs (in both positive and negative directions) were used to calculate the mutation scores. For CADD and FunSeq2, the default parameters of them are used to compute the score for each mutation. For LINSIGHT, the mutation scores were extracted from the precomputed LINSIGHT scores based on the hg19 assembly (https://github.com/CshlSiepelLab/LINSIGHT). The significant difference of mutation scores between the verified/functional mutations and the random ones is tested by Wilcoxon rank-sum test.

In general, the functional regulatory mutations exhibit higher mutation scores than those of the random occurring mutations. This characteristic allows the assessment of the prediction accuracy of various tools by applying a binary classifier on their provided mutation scores. For example, a better score will have a better binary-classification (functional versus random mutations) accuracy. By applying a cutoff value on the scores that splits the mutations to two classes (e.g., functional and nonfunctional mutations with scores greater and less than the cutoff value, respectively) then considering all possible cutoff values for the scores, the receiver operating characteristic (ROC) curves (e.g., the true positive versus the false positive rates) can be plotted for the four methods, and the plot reveals performance comparison of the binary classification (e.g., the higher the ROC curve, the better the classification accuracy). An area under this curve (AUC) represents the degree of separability for the classification i.e., the higher the AUC, the better the method is at classifying two groups. For a random-guessing classifier, the AUC of the theoretical ROC curve is 0.5.

#### Predicting mutation block-gene associations based on diverse functional genomic data

Here, bpb3 package was first applied on whole-genome somatic mutations (∼181135 of SNVs) of the second FL cohort (22 patients) obtained from ICGC public data release^57^ for identifying mutation clusters (e.g., a genomic region with at least 2 SNVs from 2 patients and the distance between the adjacent SNVs <30bp) that can be grouped into mutation blocks (e.g., a group of mutation clusters with a distance between each other <500bp; total ∼786 mutation blocks from the 22 FL patients). Then, bpb3 was used to predict differentially expressed genes (DEG; ∼5843 genes with p-value<0.05 from two samples T-test) between the FL patients and the control samples. Subsequently, the predicted 786 mutation blocks were annotated to four types genomic regions such as Gene body, TSS – from -5kbp and +1kb to TSS, TES – from -1kb and +5kb to TES, and 5’distance region – from -5kb and -1Mb to TSS based on HG19 reference genome. Other functional genomic data, such as the information of common topologically associating domains (TAD) in five human cell lines and the differential methylation regions (DMR) between FL patients and control samples, were obtained from previous publications.^35^^,^^68^ In this study, ∼248 mutation blocks are either overlapping to DMRs or associating to DEGs (e.g., a mutation block located in one of the four aforementioned genomic regions from a DEG). After requiring both the mutation block and its putative target gene (DEG) are in the same TAD^35^ for ensuring a long-distance gene regulation, ∼175 mutation blocks remaining for the further prediction of functional mutation block-gene associations.

By considering other functional genomic information such as the seven types of chromatin segmentation (or chromatin states) in human genome, a permutation enrichment test of mutation blocks (∼175 blocks) overlapping to the chromatin states was used to find the putative functional regulatory mutation block-gene associations (e.g., with ∼158 DEGs). The seven chromatin states (e.g., R, T, TSS, enhancer, CTCF, WE, PF) were obtained from earlier publications,^47^^,^^48^ which were predicted by applying machine learning methods on genome-wide chromatin features such as active/repressive histone modifications and nucleosome density et al. A brief description of the enrichment test is as follows:

(1) For *M* mutation blocks associated to a gene, a fraction of blocks (the actual fraction) located in the seven chromatin states is computed.
(2) By excluding the *M* blocks in the first step, the same number of mutation blocks were selected randomly from the initial ∼786 blocks to obtain an expected fraction of blocks located in the seven chromatin states.
(3) For each chromatin state, if the expected fraction is greater than the actual one then the chromatin state is incremented by one.
(4) After 10000 times random sampling in step 3, an expected P-value for the mutation blocks enriched in a chromatin state is calculated (e.g., P-value is equal to the number of the expected fraction greater than the actual one divided by the total number of random sampling).

If we assume that the functional regulatory mutation blocks of a gene are often enriched in either TSS or enhancer regions,^35^ then a list of putative functional regulatory mutation block-gene associations can be identified such as 51 mutation blocks associated with 12 genes from the 22 FL patients with an expected p-value <0.05 in either TSS or enhancer.

#### Abc4pwm

Affinity Based Clustering for Positions Weight Matrices - abc4pwm^40^ was developed for clustering PWMs based on their similarity. Clustering of PWMs/motifs is essential in bioinformatic data analysis. In abc4pwm, PWMs of human TFs can be assigned to their respective DBD binding families and the similarity scores of PWMs within the same DBD family are calculated, which then be used to cluster PWMs based on affinity propagation clustering.^69^ Finally, a representative motif (or pseudo-TF) of each cluster will be created by the program after clustering. For example, Figures S6 and S7 shows a representative motif of clustered PWMs. Abc4pwm is a user-friendly command line tool, more detailed description of this tool please refer to (https://github.com/abc4pwm/abc4pwm).

#### Parallel computation

In this study, all parallel calculations were done in SAGA supercomputer with 10 parallel processes. SAGA is provided by Hewlett Packard Enterprise - Apollo 2000/6500 Gen10 (Intel Xeon-Gold 6138 2.0 GHz/6230R 2.1 GHz, 200 standard compute nodes with 40 cores and 192 GiB memory each), which is placed at NTNU in Trondheim but we are physically located in Oslo Norway. Thus, the work can be seen as an application of cloud computing in the evaluation of bpb3 performance. More information of high-performance computing please refer to bpb3 online documentation (https://bpb3.github.io/bpb3/). The cost of CPU hours, memory usages, and data storage are supported by NRIS – Norwegian research infrastructure services through project number NN4605K and NS4605K.

### Quantification and statistical analysis

Statistical analyses were performed using Python or the designed bpb3 Python package. More information please refer to bpb3 online documentation (https://bpb3.github.io/bpb3/).

## Data Availability

• The bpb3 Python package, tutorial script, and all other data have been deposited on our GitHub repository https://github.com/junbaiw/bpb3 and project homepage https://bpb3.github.io/bpb3/, which are publicly available as of the date of publication. • Additional information required to reanalyze the data reported in this paper is available from the lead contact upon request.
