# Supplementary material for: Identifying functional regulatory mutation blocks by integrating genome sequencing and transcriptome data
Source: iScience. 2023 Jul 3;26(8):107266. doi: 10.1016/j.isci.2023.107266 (PMC10371843; doi:10.1016/j.isci.2023.107266)
Supplement: Document S1. Figures S1–S7 and Tables S1–S4 [file mmc1.pdf]

iScience, Volume *26*

Supplemental information

Identifying functional regulatory mutation blocks by integrating genome sequencing and transcriptome data

Mingyi Yang, Omer Ali, Magnar Bjørås, and Junbai Wang

Supplementary Figures S1-S7:

Figure S1. Accuracy and time consumption for predicting TF binding affinity changes of 67 SNPs based on three types of calculations, related to Table 1.

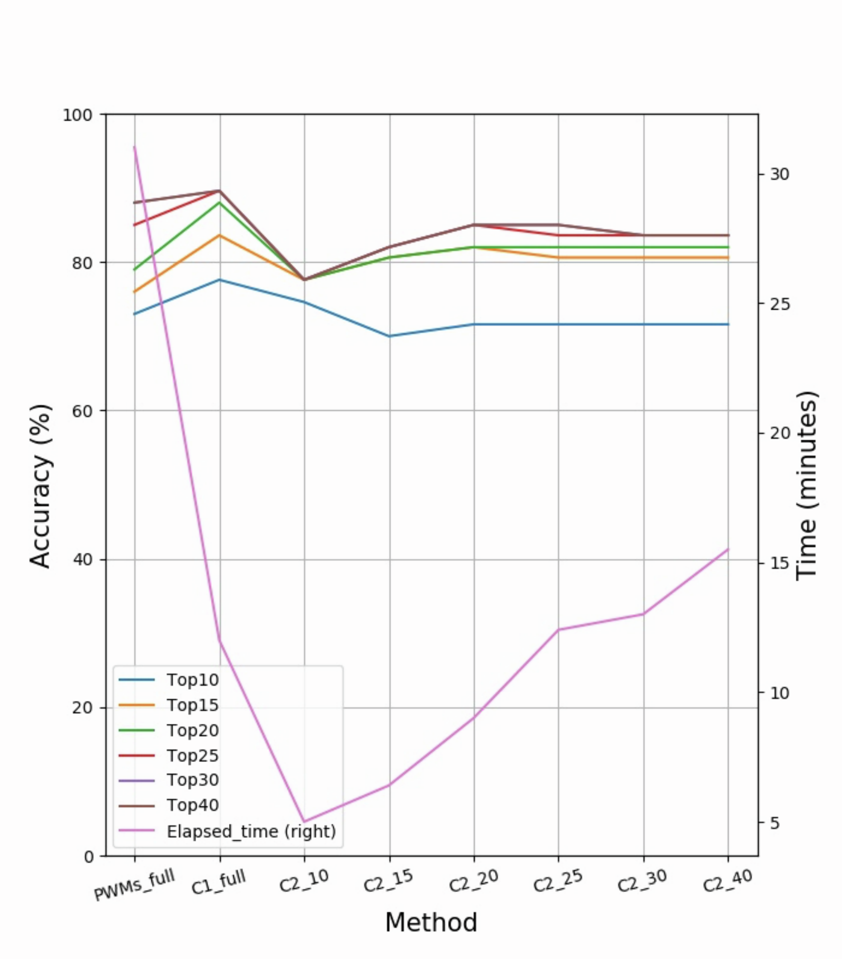

The Top10, 15, 20, 25, 30, and 40 represent the accuracy of predicted TF binding affinity changes due to SNPs in the top 10, 15, 20, 25, 30, and 40 of predicted TFs respectively. Elapsed_time means the CPU times that were used in prediction. The PWMs_full and C1_full indicate predictions based on the full 1772 human TFs and clustered PWMs level 1 (e.g., ~736 pseudo-human TFs), respectively. C2_10, _15, _20, _25, _30, and _40 represent predictions from C2 approach (clustered PWMs level 2) by selecting the top 10, 15, 20, 25, 30, and 40 TFs from C1, respectively.

Figure S2. Significant TF binding affinity changes (C1fC2_25 by two-level approach) in the promoter region of BCL2 where a patient-specific mutation block is predicted from 14 follicular lymphoma patients, related to Figures 3 and 4.

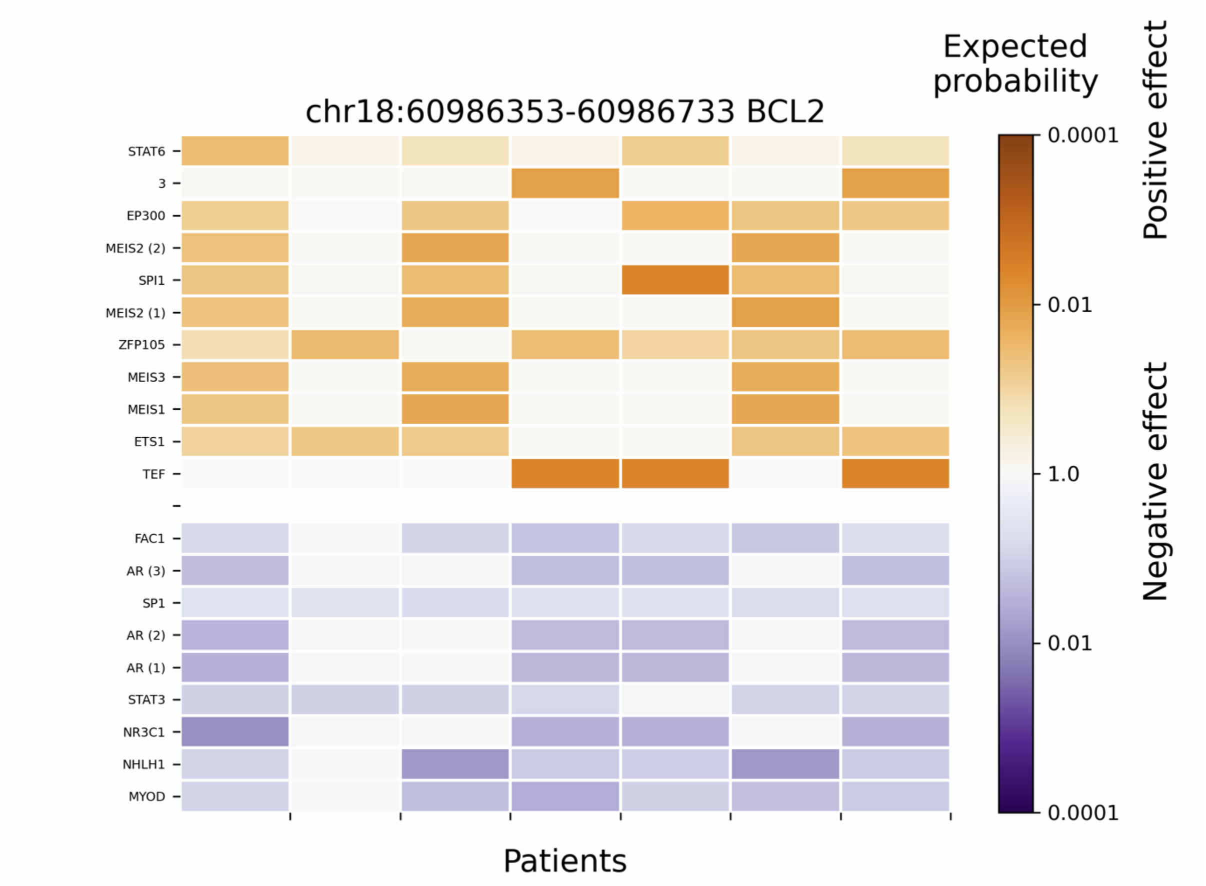

Here, the output of top 25 pseudo human TFs from clustered PWMs level 1 foreground calculation (C1_full method) were used as input in the clustered PWMs level 2 (C2 method) – C1fC2_25, to predict TFs with significant binding affinity changes in mutation blocks at promoter regions (e.g., +/-1000bp of TSS). The row labels are the TF name, and the column labels are patients with a regulatory mutation block (chr18:60986353-60986733) at the promoter region of BCL2. TF names are repeated when several alternative PWMs for a single TF are significantly affected. The color encodes the expected probability that the TF will be affected by random mutations as strongly as by the patient mutations (median size of significant blocks is 457bp), on the logarithmic scale. The positive and negative affinity changes are colored orange and blue, respectively. TFs with very low expression (RPKM < 0.03) were filtered out. Only TFs with significant changes ($P < 0.01$) across all patients are shown.

Figure S3. Significant TF binding affinity changes (C1fC2_25 by two-level approach) in the promoter region of TERT where a patient-specific mutation block is predicted from 263 melanoma patients, related to Figures 5 and 6.

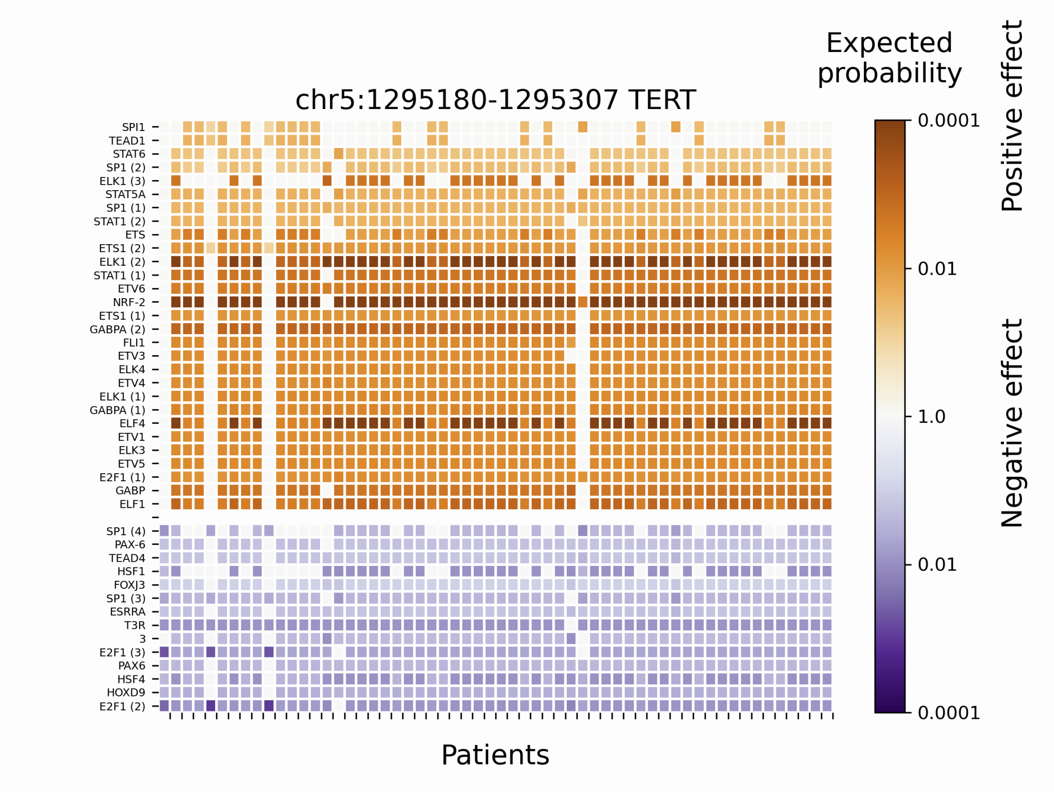

Here, the top 25 pseudo human TFs from clustered PWMs level 1 foreground calculation (C1_full method) were used by clustered PWMs level 2 (C2 method) – C1fC2_25, to predict TFs with significant binding affinity changes in mutation blocks at the promoter regions (e.g., +/-1000bp of TSS). The row labels are the TF name, and the column labels are patients with a regulatory mutation block (chr5:1295180-1295307) at the promoter region of TERT. TF names are repeated when several alternative PWMs for a single TF are significantly affected. The color encodes the expected probability that the TF will be affected by random mutations as strongly as by the patient mutations (median size of significant blocks is 144bp), on the logarithmic scale. The positive and negative affinity changes are colored orange and blue, respectively. TFs with very low expression (RPKM < 0.03) were filtered out. Only TFs with significant changes (Bonferroni-adjusted p-value < 0.001) across all patients are shown. ELK1, ELK3, ELK4, ETS1, ETV3, ETV4, ETV5, ELF1, ELF4, and GABPA are belong to ETS-related families.

Figure S4. Cumulative accuracy of predicted TF rankings for 67 verified mutations, related to Figure 7.

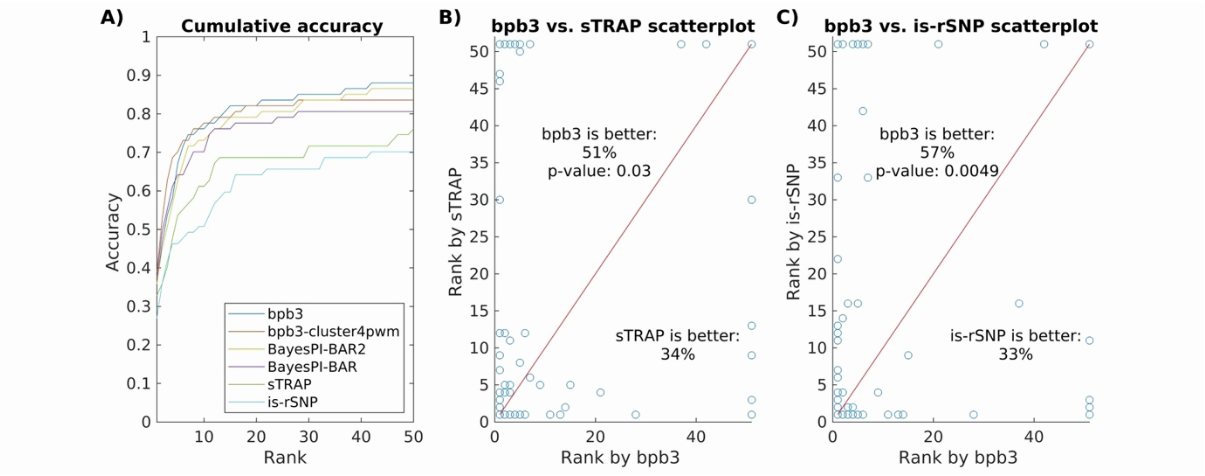

(A) Cumulative accuracy plot by tools of bpb3, BayesPI-BAR, sTRAP and is-rSNP respectively. The X-axis is the top ranked TFs from the prediction, the Y-axis is the percentage of true mutation prediction by the corresponding top ranked TFs. Here, the direction of TF binding affinity changes is not considered.
(B) Scatter plot of the predicted ranking for the true target TF of mutations (e.g., bpb3 versus sTRAP), where the maximum rank is 50. Mutations above the diagonal red line (X = Y) are ranked better by bpb3, but those below the diagonal red line are ranked better by sTRAP. The p-value is the Wilcoxon signed-rank test after comparing the paired ranks from the two methods.
(C) The same plot as in Figure S5B but for bpb3 versus is-rSNP.

Figure S5. Significant TF binding affinity changes at block_4_18_60987833_60988772 when applying bpb3 on the second FL cohort data (22 patients) by using a set of predefined genomic regions, related to Figure 9.

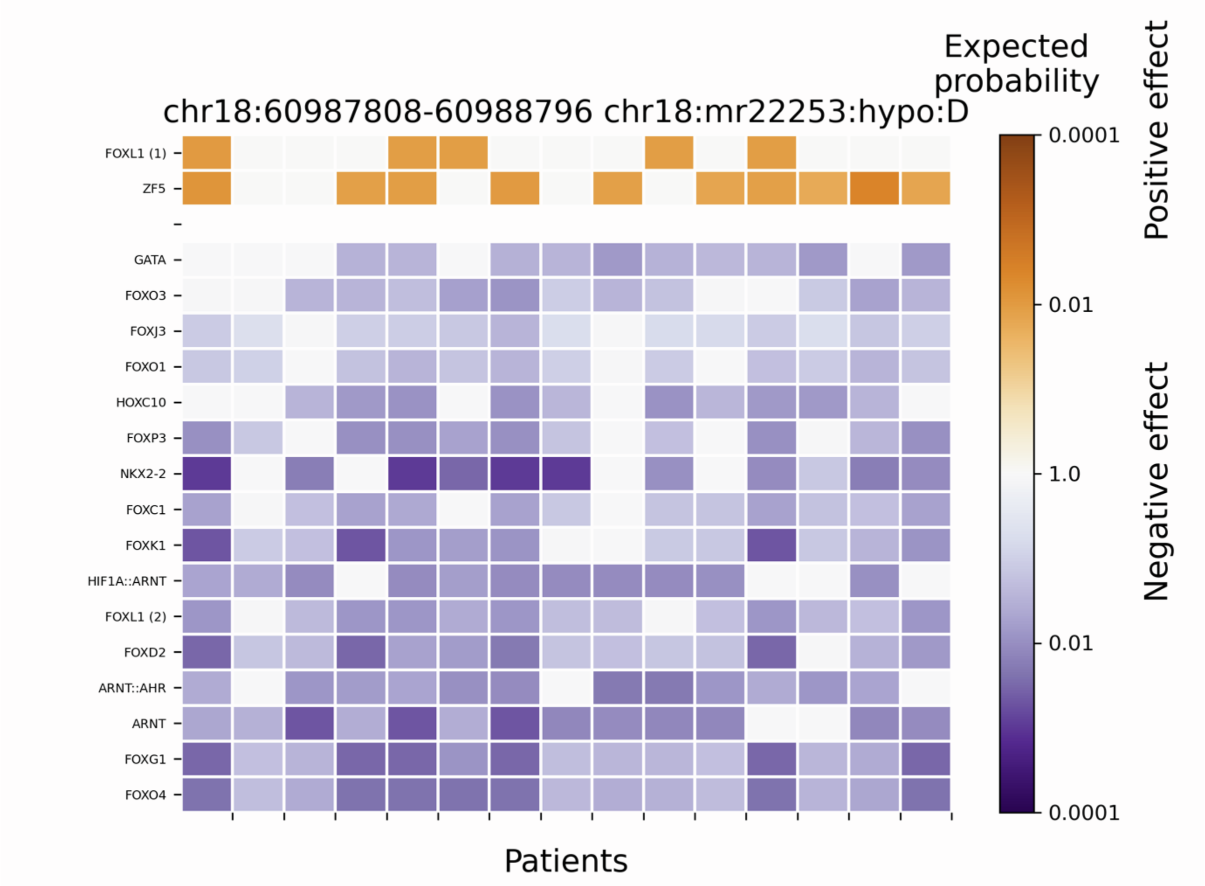

Around 1772 human TF PWMs were used (PWMs_full method) to evaluate their significant binding affinity changes in a significant mutation block (block_4_18_60987833_60988772; Bonferroni-adjusted P<0.001) that detected at a set of predefined genomic regions (e.g., predicted differential DNA methylation regions – DMR overlapping to enhancers in 14 FL patients[1]) by applying bpb3 on the second FL cohort data (22 patients). The row labels are the TF names, and the column labels are patients with a regulatory mutation block (chr18: 60987808-60988796) that overlapping/nearby DMRs and enhancers. The TF names are repeated when several alternative PWMs for a single TF are significantly affected. The color encodes the expected probability that the TF binding affinity will be affected on the logarithmic scale. The positive and negative affinity changes are colored orange and blue, respectively. TFs with very low expression (RPKM < 0.03) were filtered out. Only TFs with significant changes (Bonferroni-adjusted p-value < 0.05) across all patients are shown.

Figure S6. An example of representative motif for a cluster of PWMs in bZIP DBD family, related to STAR Methods.

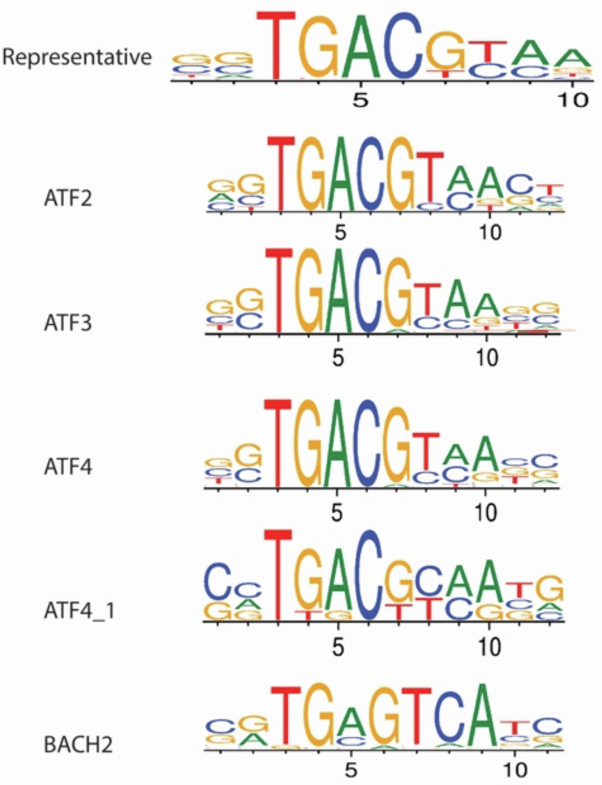

Here, there are five PWMs in a cluster (ATF2, ATF3, ATF4, ATF4_1 and BACH2) from bZIP family. A representative motif of this cluster is shown on the top of the figure. Abc4pwm generates PWM cluster as well as produces the representative motif for the cluster.

Figure S7. **An example of application of clustered PWMs (or representative motifs) in bpb3 data analysis, related to STAR Methods.**

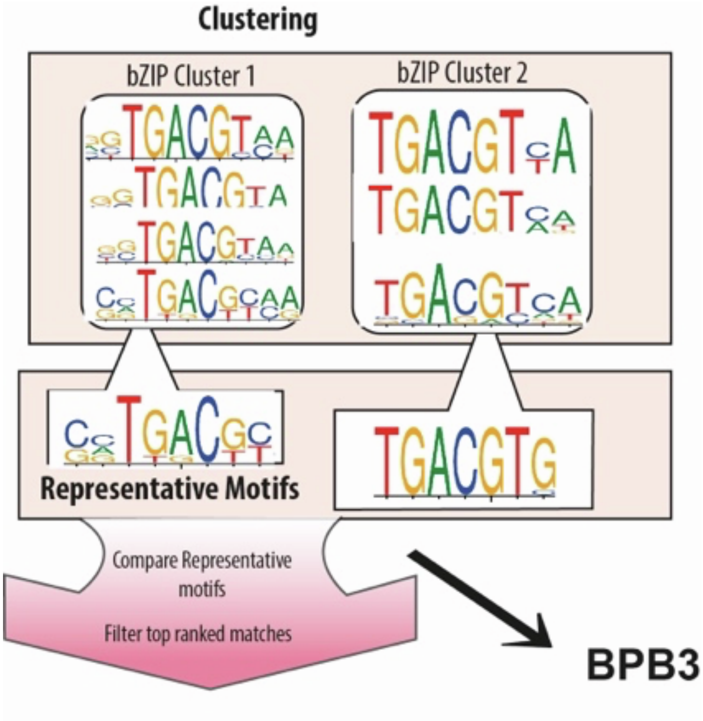

Supplementary Tables S1-S4:

Table S1. Elapsed time for predicting significant TF binding affinity changes in mutation blocks of cancer patient cohorts by WGS and RNA-seq data integration, related to Figure 2.

Method	Elapsed time FL14 (4 blocks)	Elapsed time second cohort (12 blocks)	Elapsed time skin cancer (12 blocks)
PWMs_full (foreground)	2hr 31mt	7hr 25mt	7hr 10mt
PWMs_full (background)	1hr 51mt	3hr 24mt	1hr
C1_full (foreground)	1hr	3hr 5mt	3hr 1mt
C1_full (background)	1hr 15mt	2hr 3mt	30mt
C2_15	1hr	2hr 11mt	2hr 1mt
C2_20	1hr 8mt	2hr 26mt	2hr 30 mt
C2_25	1hr 14mt	2hr 42mt	3hr 29mt

This table shows CPU time used in analyzing WGS and RNA-seq data from cancer patients by using different combinations of the pipeline. All calculations were done in the same computer with 10 parallel processes. FL14 are data from published 14 follicular lymphoma samples, which detected 4 significant mutation blocks. Second cohort is an independent follicular lymphoma patient cohort downloaded from ICGC, which identified 12 significant mutation blocks. Skin cancer is a melanoma cancer patient cohort downloaded from ICGC, which predicted 12 significant mutation blocks. The PWMs_full and C1_full indicate predictions based on the full 1772 human TFs and the clustered PWMs level 1 (e.g., ~736 pseudo human TFs), respectively. Foreground and background represent the calculation of TF binding affinity changes from input patient data and the estimation of TF binding affinity changes in foreground and background model, respectively, both are needed in identifying significant TF binding affinity changes in cancer mutation blocks. C2_15, _20, and _25 represent predictions from C2 (clustered PWMs level 2) by selecting the top 15, 20, 25 pseudo-TFs from C1, respectively, where both foreground and background calculations were performed in order to predict TFs with significant binding affinity changes in the mutation blocks.

Table S2. Predicted mutation blocks by applying bpb3 package on the second follicular lymphoma (FL) cohort (22 patients) with predefined genomic regions in chr18 (e.g., differential DNA methylation region – DMR overlapping to enhancers), related to Figure 9.

block_id	chrom	start_pos	end_pos	number_of_mutations	number_of_patients	mutation_distribution	region_names
***block_3_18_60983893_60987060**	18	60983868	60987084	399	18	22,36,15,6,25,24,5,40,33,16,38,7,37,15,1,37,20,22	chr18:mr22250:hyper:D,chr18:mr22251:hyper:D,chr18:mr22252:hypo:D,chr18:mr22253:hypo:D
***block_4_18_60987833_60988772**	18	60987808	60988796	180	15	6,6,9,5,8,9,11,16,8,14,11,21,13,37,6	chr18:mr22253:hypo:D
***block_0_18_60805516_60805690**	18	60805491	60805714	11	5	2,1,1,6,1	chr18:mr22142:hypo:D,chr18:mr22148:hyper:D,chr18:mr22149:hypo:D
**block_2_18_60875928_60876041**	18	60875903	60876065	7	4	1,1,3,2	chr18:mr22186:mix:D,chr18:mr22187:hyper:D,chr18:mr22188:hypo:D,chr18:mr22189:hypo:D
**block_1_18_60809575_60809602**	18	60809550	60809626	5	3	1,2,2	chr18:mr22148:hyper:D,chr18:mr22149:hypo:D,chr18:mr22150:hypo:D,chr18:mr22151:hypo:D

Here, “block_id” marked by * means significant mutation blocks identified by bpb3 package (Bonferroni-adjusted P-value <0.001). “chrom”, “start_pos”, “end_pos” represent chromosome name, chromosome start position, and chromosome end position of a mutation block, respectively, where 25bp flank regions are added to the two-side of original mutation block. “number_of_mutations” and “number_of_patients” are the number of mutations and patients in a mutation blocks, respectively. “mutation_distribution” shows the number of mutations of each patient in a mutation block. “region_names” are DMRs overlapping/within 5000bp to a mutation block, where the DMRs information were obtained from a previous publication in 14 FL patients [1].

Table S3. Predicted regulatory mutation block-gene associations by bpb3 package in the second follicular lymphoma (FL) cohort (22 patients) with an enrichment test on seven chromatin states, related to Figure9.

Gene name	R	E	T	TSS	PF	CTCF	WE	Mean scores	Block ID
BCL7A	0.00	2.44	0.00	2.33	1.45	0.00	0.00	0.77	block_396_12_122460749_122461751
									block_395_12_122458889_122459429
IGLL5	3.04	1.34	0.54	0.56	0.00	5.00	0.00	0.69	block_772_22_23227612_23228303
									block_771_22_23222972_23223865
									block_768_22_23055160_23055248
									block_765_22_22724235_22724262
									block_777_22_23242054_23242183
									block_773_22_23229502_23229512
									block_774_22_23230017_23232858
									block_767_22_23029525_23029738
									block_769_22_23198213_23199061
									block_776_22_23234417_23235623
									block_775_22_23233381_23233630
									block_766_22_23028899_23028902
									block_770_22_23204419_23204758
									block_764_22_22516837_22516899
PAX5	1.07	5.00	0.18	2.49	0.00	0.00	0.00	0.69	block_450_9_37371680_37371942
									block_447_9_37026296_37026901
									block_446_9_37024575_37025597
									block_452_9_37387557_37387560
									block_451_9_37383929_37384082
BCL2	1.23	5.00	0.48	3.68	2.51	0.91	1.09	0.66	block_493_18_60987833_60988858
									block_491_18_60875928_60876041
									block_489_18_60806367_60806689
									block_492_18_60983828_60987060
									block_490_18_60809479_60809602
									block_488_18_60805267_60805690
RGL4	2.38	0.87	0.18	2.20	0.00	3.29	0.00	0.64	block_771_22_23222972_23223865
									block_772_22_23227612_23228303
									block_768_22_23055160_23055248
									block_778_22_23244238_23244255
									block_777_22_23242054_23242183
									block_773_22_23229502_23229512
									block_780_22_23278583_23278637
									block_769_22_23198213_23199061
									block_779_22_23247490_23247522
									block_781_22_23282478_23282570
									block_770_22_23204419_23204758
BCR	2.10	2.04	0.23	2.49	0.00	5.00	0.00	0.61	block_771_22_23222972_23223865
									block_772_22_23227612_23228303
									block_782_22_23523796_23523824
									block_778_22_23244238_23244255
									block_768_22_23055160_23055248
									block_765_22_22724235_22724262
									block_777_22_23242054_23242183
									block_773_22_23229502_23229512
									block_780_22_23278583_23278637
									block_767_22_23029525_23029738
									block_769_22_23198213_23199061
									block_766_22_23028899_23028902
									block_779_22_23247490_23247522
									block_781_22_23282478_23282570
									block_770_22_23204419_23204758
BCL6	0.28	3.15	0.00	3.21	2.66	0.00	0.00	0.60	block_632_3_187463866_187463886
									block_633_3_187661049_187661142
									block_631_3_187461042_187463057
									block_630_3_187460192_187460221
RHOH	0.82	3.09	0.24	2.85	1.15	0.00	1.27	0.59	block_269_4_40196468_40196491
									block_268_4_40175411_40175428
									block_271_4_40201021_40201366
									block_270_4_40198815_40200489
BACH2	0.62	3.51	0.00	3.51	0.00	0.00	0.00	0.52	block_745_6_91007022_91007026
									block_743_6_91005079_91005086
									block_744_6_91005644_91006035

**LPP**	0.58	1.35	1.86	0.41	0.98	0.00	0.00	0.47	block_637_3_188471653_188471734<br>block_635_3_187959947_187959964<br>block_634_3_187958417_187959000<br>block_632_3_187463866_187463886<br>block_633_3_187661049_187661142<br>block_636_3_188299471_188299500
**CIITA**	0.42	0.95	0.00	2.26	0.00	1.39	0.00	0.36	block_207_16_10971520_10973148<br>block_208_16_10973678_10973814
**IL4R**	0.07	0.00	0.00	2.27	0.00	0.00	1.61	0.25	block_210_16_27325675_27325765<br>block_211_16_27326880_27326898

There are 12 genes and 51 mutation blocks passed the enrichment test for mutation block—gene associations in seven chromatin states (e.g., with expected P-values <0.05 or -log10(P-values)>1.3 in either TSS or Enhancer regions). The seven types of chromatin segmentation of human genome[2] (or chromatin states) are R, T, E, TSS, WE, CTCF, and PF that represent the predicted repressed/low activity region, transcribed region, enhancer, promoter region/transcription start site, weak enhancer/open chromatin region, CTCF enriched element, and promoter flanking region, respectively. The mean score represents the mean feature scores of mutation blocks, which was calculated based on four features of mutation blocks (e.g., the number of patients affected by mutation blocks, DMR significance, P-value to DEG, and the weighted genomic feature of a mutation block). Please refer to previous paper[1] for more detailed description of this feature score. Here, both BCL2 and BCL6 with their previously published regulatory mutation blocks[3, 4] (highlighted by yellow color in the table) are recovered in this analysis.
